# Fixed-parameter tractable sampling for RNA design with multiple target structures

**DOI:** 10.1186/s12859-019-2784-7

**Published:** 2019-04-25

**Authors:** Stefan Hammer, Wei Wang, Sebastian Will, Yann Ponty

**Affiliations:** 1 Dept. Computer Science, and Interdisciplinary Center for Bioinformatics, Univ. Leipzig, Härtelstr. 16-18, Leipzig, D-04107 Germany; 2 Dept. Theoretical Chemistry, Univ. Vienna, Währingerstr. 17, Wien, A-1090 Austria; 3 Bioinformatics and Computational Biology Research Group, Univ. Vienna, Währingerstr. 17, Wien, A-1090 Austria; 4 CNRS UMR 7161 LIX, Ecole Polytechnique, Bat. Alan Turing, Palaiseau, 91120 France

**Keywords:** RNA multi-target design, RNA secondary structure, Multi-dimensional Boltzmann sampling, *#*P-hardness of RNA design

## Abstract

**Background:**

The design of multi-stable RNA molecules has important applications in biology, medicine, and biotechnology. Synthetic design approaches profit strongly from effective in-silico methods, which substantially reduce the need for costly wet-lab experiments.

**Results:**

We devise a novel approach to a central ingredient of most in-silico design methods: the generation of sequences that fold well into multiple target structures. Based on constraint networks, our approach  supports generic Boltzmann-weighted sampling, which enables the positive design of RNA sequences with specific free energies (for each of multiple, possibly pseudoknotted, target structures) and GC-content. Moreover, we study general properties of our approach empirically and generate biologically relevant multi-target Boltzmann-weighted designs for an established design benchmark. Our results demonstrate the efficacy and feasibility of the method in practice as well as the benefits of Boltzmann sampling over the previously best multi-target sampling strategy—even for the case of negative design of multi-stable RNAs. Besides empirically studies, we finally justify the algorithmic details due to a fundamental theoretic result about multi-stable RNA design, namely the #P-hardness of the counting of designs.

**Conclusion:**

introduces a novel, flexible, and effective approach to multi-target RNA design, which promises broad applicability and extensibility.

Our free software is available at: https://github.com/yannponty/RNARedPrint Supplementary data are available online.

**Electronic supplementary material:**

The online version of this article (10.1186/s12859-019-2784-7) contains supplementary material, which is available to authorized users.

## Background

Synthetic biology strives for the engineering of artificial biological systems, promising broad applications in biology, biotechnology and medicine. Centrally, this requires the design of biological macromolecules with highly specific properties and programmable functions. RNAs are particularly well-suited tools for rational design targeting specific functions [1]: on the one hand, RNA function is tightly coupled to the formation of secondary structure, as well as changes in base pairing propensities and the accessibility of regions, e.g. by burying or exposing interaction sites [2]; on the other hand, the thermodynamics of RNA secondary structure is well understood and its prediction is computationally tractable [3]. Thus, in rational design approaches, structure can serve as effective proxy for, the ultimately targeted, catalytic or regulatory functions [4].

The function of many RNAs depends on their selective folding into one or several alternative conformations. Classic examples include riboswitches, which adopt different stable structures upon binding a specific ligand. Riboswitches have been a popular application of rational design [5, 6], partly motivated by their capacity to act as biosensors [7], which suggests them for biotechnological applications. In particular due to the kinetic coupling of RNA folding with RNA transcription, RNA families can feature alternative, evolutionarily conserved, transient structures [8], which are essential for the formation of their functional structures. More generally, simultaneous compatibility to multiple structures is a relevant design objective for engineering kinetically controlled RNAs, finally targeting prescribed folding pathways. Thus, advanced applications of RNA design often target multiple structures, additionally aiming at other features, such as specific GC-content (GC%) [9] or the presence/absence of functionally relevant motifs, either anywhere or at specific positions [10]; these objectives motivate flexible computational design methods.

Many computational methods for RNA design follow the “generate-and-optimize” strategy: *seed* sequences are randomly generated and then optimized. While the quality of the seeds was found to be performance-critical for such RNA design methods [11], random seed generation can improve the prospect of subsequent optimizations and increases the diversity across designs [9]. For single-target approaches, INFO-RNA [12] could significantly improve the success rate over RNAinverse [13], by starting its local search from the minimum energy sequence for the target structure. Since this strategy typically designs sequences with unrealistically high GC%, more recent approaches like antaRNA [14] and IncaRNAtion [9] explicitly control GC%; the latter applying adaptive sampling.

The available methods for multi-target RNA design [15–18] all follow the same overall generate-and-optimize strategy. Faced with the complex constraints due to the multiple targets, early methods such as Frnakenstein [15] and Modena [17] do not even attempt to sample sequences systematically from a controlled distribution, but rely on ad-hoc generation strategies. Recently, the approach RNAdesign [16], coupled with local search in  [18], solved the problem of sampling seeds from the *uniform* distribution for multiple target structures. RNAdesign adopts a graph coloring perspective, assigning nucleotide symbols (like “colors”) to the sequence positions, such that compatible nucleotides are assigned to the ends of each base pair. Initially, the method decomposes the graph hierarchically and then *precomputes* the number of valid sequences within each subgraph. The decomposition is then reinterpreted as a decision tree to perform *stochastic backtracking*, inspired by Ding and Lawrence [19]. Uniform sampling is achieved by choosing individual nucleotide assignments with probabilities derived from the subsolution counts. While, due to its decomposition strategy, RNAdesign performs much better than the theoretical bound of *O*(4^*n*^), no attempts were made to characterize or justify its—still exponential—complexity; leaving important theoretical questions of the complexity of counting and uniform sampling open. As well, the  approach is specialized to uniform sampling, which limits its direct extensibility. Substantial improvements of multi-target sampling thus require a systematically redesigned approach. To enable a fundamentally broader range of applications in extensions of the sampling method, we build our approach, from the start, on established concepts in computer science.

### Contributions

As central contribution, we provide a systematic and flexibly extensible technique for sampling that targets multiple versatile features. For the sake of clarity, we introduce this method specialized to the sampling of RNA sequences that have specific energies for multiple structures and specific GC%. In this way, we address the positive design of RNA sequences. Positive design is contrasted to the often desirable negative design of RNAs, which optimizes the stability of the target structures *in relation to all other potential structures*. Remarkably, the even more complex task of negative design immediately benefits from positive design (Additional file 1: Section A), which provides an initial motivation to study the positive design problem by itself.

**Fig. 1:**
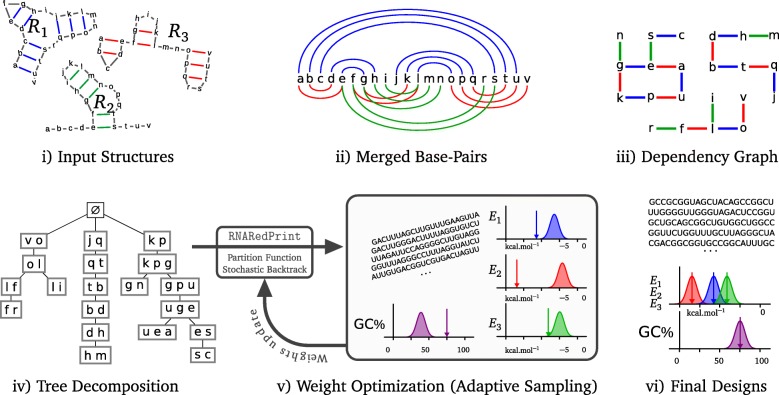
General outline of . From a set of target secondary structures (**i**), base pairs are merged (**ii**) into a (base pair) dependency graph (**iii**) and transformed into a tree decomposition (**iv**). The tree is then used to compute the partition function, followed by a Boltzmann sampling of valid sequences (**v**). An adaptive scheme learns weights to achieve targeted energies and GC% (arrows), leading to the production of suitable designs (**vi**). Note that for simplicity, we assume in this figure that only dependencies between the ends of base pairs are considered to evaluate the energies of structures. Our computations based on a more complex energy model, which considers energy contributions of base pair stacks, require additional dependencies

Figure 1 summarizes our generic framework, which enables this targeted sequence generation based on multi-dimensional Boltzmann sampling. *Algorithmically*, we originally contribute dynamic programming (DP) algorithms, based on the concept of *tree decomposition*, to compute partition functions and sample sequences from the Boltzmann distribution. Generally, tree decompositions are data structures that capture the specific dependencies of a problem instance (here, the dependencies between sequence positions induced by the target structures), such that they can guide the efficient processing by DP algorithms. Building on this principle, the complexities of our algorithms depend exponentially on a specific property of the tree decomposition, called the *treewidth*. Thus, it is essential for the applicability of our approach that—by appropriate design choices—we can keep this parameter low for typical instances. For any fixed value of the treewidth, the complexity scales only linearly with the size of designed sequences and the number of targeted structures, i.e. our algorithms are *fixed-parameter tractable (FPT)*.

Remarkably, we could show that it is not possible to find a better, efficient method for sampling (unless P=NP), since the underlying counting problem is #P-hard. The practical relevance of this theoretical result is that it rules out substantially better sampling techniques. Even when using improved sampling methods, there will always remain an upper limit on the (in practice) tractable number and heterogeneity of structures, the complexity of the directly treatable energy model, and the number and complexity of additional constraints that could be considered in future sampling-based applications. Technically, this result relies on a surprising bijection between valid sequences and independent sets of a bipartite graph, the latter being the object of recent breakthroughs in approximate counting complexity [20, 21].

Due to the generality of our method, we can moreover strongly limit the treewidth in practice by using state-of-the-art tree decomposition algorithms. By evaluating sequences in a specialized weighted constraint network, we support—in principle—arbitrary complex constraints and energy models, notably subsuming the commonly used RNA energy models. Moreover, we describe an *adaptive sampling* strategy to control the free energies of the individual target structures and GC%.

We observe that targeting realistic RNA energies in the Turner RNA energy model works well by performing sampling based on a simplified RNA energy model, which induces much lower treewidth than the Turner model. This result is essential for the applicability of our method, since it allows to combine high efficiency (by keeping the treewidth low) with sufficient accuracy to precisely target realistic Turner energies.

Eventually, our proof-of-concept results on a comprehensive multi-target RNA design benchmark [17] suggest that our sampling strategy well supports designing biologically relevant RNAs for multiple targets.

## Methods

The main computational problem addressed in this work is the positive design of RNA sequences for multiple target structures; more specifically, the generation of sequences over the alphabet *Σ*={A,C,G,U}, such that the sequences feature a given GC*%*, and have prescribed energies for a set of target secondary structures. Here, these desired sequence properties are modeled as constraints on the values of *features*, which are functions of the sequence that are expressed as sums over real-valued *contributions*. Each contribution depends on the nucleotides at—typically few—specific sequence positions.

To generate diverse design candidates, we randomly generate sequences from a Boltzmann distribution. The probability of a sequence then depends on its features (e.g. the energies of the target structures), and the weight of each feature (which influences its distribution). Sampling from the (multi-feature) Boltzmann distribution requires to compute corresponding partition functions, such that we can draw sequences with probabilities proportional to their Boltzmann weight. On this basis, we can finely calibrate the weights, to maximize the probability that sampled sequences meet the desired target values for each feature. Together with a final rejection step this results in an effective procedure for generating highly specific sequences.

### Problem statement

Let us consider a set of *k* (secondary) structures $\mathcal {R} = \{R_{1},\dots, R_{{k}}\}$, each abstracted as a set of base pairs, and *m*≥*k* features *F*_1_,…,*F*_*m*_, typically representing the energies of the structures and additional sequence properties, associated with weights *π*_1_,…,*π*_*m*_ in $\mathbb {R}^{+}$. Our goal is to sample sequences *S* (which satisfy the base pairing rules for all structures) from the Boltzmann distribution defined by

1 $$  \mathbb{P}(S\mid\pi_{1},\dots,\pi_{{m}}) \propto \prod_{1\leq \ell\leq {m}}\!\! \pi_{\ell}^{-F_{\ell}(S)}  $$

The workhorse of our approach is the fixed-parameter tractable computation of feature-dependent partition functions over sequences, namely partition functions of the form

2 $$  Z_{{\pi_{1},\dots,\pi_{{m}}}} = \sum_{S\in\Sigma^{n}} \prod_{1\leq \ell\leq {m}} \pi_{\ell}^{-F_{\ell}(S)},  $$

for specific weights *π*_1_,…,*π*_*m*_.

### Expressing GC%-content, sequence validity and energies as features

Formally, we define a *feature F* as a function on sequences, whose value is obtained by summing over an associated set of *contributions*. Each contribution *f* takes values in $\mathbb {R}\cup \{+\infty \}$, and depends on the nucleotides assigned to a restricted set of positions, namely its *dependencies*, denoted dep(*f*), such that

$$F(S) =\!\!\sum_{\substack{{f} \text{ contribution of } {F},\\{\text{ dep }(f)=\{x_{1},\ldots,x_{p}\}}}}\!\! f\left(\left\{\substack{x_{1}\mapsto S_{x_{1}}\\\cdots\\x_{p}\mapsto S_{x_{p}}}\right\}\right). $$

Here, since $\text {dep}(f)=\{x_{1},\ldots,x_{p}\}, \left \{\substack {x_{1}\mapsto S_{x_{1}}\\\cdots \\x_{p}\mapsto S_{x_{p}}}\right \}$denotes the assignment, that assigns the respective nucleotides $S_{x_{q}}$ (1≤*q*≤*p*; *p*=|dep(*f*)|) to the positions *x*_*q*_ in dep(*f*).

The GC*%* can be simply expressed using *n* contributions $f^{\textsf {GC}}_{i}$, each depending only on position *i*∈[1,*n*], i.e. $\text {dep}(f^{\textsf {GC}}_{i})=\{i\}$, such that

$$f^{\mathsf{GC}}_{i}(\{i\mapsto c\})= \left\{\begin{array}{ll} -1&\text{if}\ c={\mathsf{G}}\ \text{or}\ {\mathsf{C}}\\ 0&\text{ otherwise}. \end{array}\right. $$

By summing $f^{\textsf {GC}}_{i}(\{i\mapsto S_{i}\})$ over the whole sequence (*i*=1,…,*n*), one simply counts the occurrences of G and C.

To start with a simple example of evaluating the energy of sequences by features, let us explain how they are used to count the number of *valid sequences*, i.e. sequences inducing only base pairs in $\mathcal {B}:=\{\{{\textsf {A}},{\textsf {U}}\}, \{{\textsf {G}},{\textsf {C}}\}, \{{\textsf {G}},{\textsf {U}}\}\}$. Consider a feature *F*^BP^ composed of contributions $f^{\texttt {BP}}_{i,j}$, for each base pair (*i*,*j*) occurring in some structure, such that

$$f^{\mathsf{BP}}_{i,j}\left(\left\{\substack{i\mapsto a\\j\mapsto b}\right\}\right)= \left\{\begin{array}{ll} 0&\text{if}\ \{a,b\}\in\mathcal{B} \\+\infty&\text{otherwise}. \end{array}\right. $$

The value of *F*^BP^ is 0 for any valid sequence, and +*∞* as soon as some non canonical base pair is created. For any associated weight *π*_BP_>1, the contribution of a valid sequence is $\pi _{\textsf {BP}}^{0} = 1$, and the contribution of an invalid sequence is $\pi _{\textsf {BP}}^{+\infty } = 0$, so that Eq. 2 (when restricted to *F*^BP^) simply counts the number of valid sequences.

Energy models for structure prediction vary considerably, yet can always be expressed as sums over contributions associated with local structural motifs (base pairs, base pair stacks, loops, …) under a certain nucleotide assignment. Energy models can thus be captured generically by introducing, for each motif $\frak {m}$ occurring in a target structure, a contribution $f_{\frak {m}}$, taking a specific value for each assignment of nucleotides to its positions $\text {dep}(f_{\frak {m}})$. For instance, the contribution of a *base pair stack*, consisting of two pairs (*i*,*j*) and (*i*+1,*j*−1), can be captured by the introduction of a function $f^{\textsf {Stack}}_{\substack {i,j}}$ such that $\text {dep}\left (f^{\textsf {Stack}}_{\substack {i,j}}\right) = \{i,i+1,j-1,j\}$. We refer to energy models that consider the contributions of all base pair stacks (and thus introduce the corresponding dependencies) collectively as the *stacking energy model* (briefly, *stacking model*).

### Dependency (hyper)graph, tree decomposition and treewidth

In order to compute the partition function of Eq. 2, and thus sample in a well-defined way, one must consider dependencies induced by the complete set of contributions

$$\mathcal{F} := \bigcup_{\ell}\{f\mid f\text{contribution of}\ F_{\ell}\}. $$

In the simplest case, this set captures the requirement of canonical base pairing for each structure. To express this, let us define the *base pair dependency graph*$G_{\mathcal {R}}$ as the graph with nodes {1,…,*n*} and edges $\bigcup _{\ell \in [1,k]} R_{\ell }$.

Since $\mathcal {F}$ defines potentially more complex dependencies, which can relate more than two positions, in general its dependencies cannot be represented by a graph. Instead, this requires a structure known as *hypergraph*, which consists of vertices (here, the sequence positions) connected by *hyperedges*, which are arbitrary sets of vertices. In this way, hypergraphs generalize undirected graphs where each edge is a set of exactly two vertices. The *dependency (hyper)graph induced by*$\mathcal {F}$ is then defined as the hypergraph $G_{\mathcal {F}}=(V, H)$ on sequence positions *V*={1,…,*n*} by interpreting the dependencies as hyperedges, i.e. $H =\{\text {dep}(f)\mid f\in \mathcal {F}\})$.

Let us finally define the *tree decomposition* of the graph $G_{\mathcal {F}}$, a fundamental ingredient of our algorithms, which also determines their efficiency (most importantly, via its property called treewidth).

#### **Definition 1**

(Tree decomposition and treewidth) Let *G*=(*X*,*E*) be a (hyper)graph with nodes in *X* and (hyper)edges in *E*. A *tree decomposition* of *G* is a pair (*T*,*χ*), where *T* is an unrooted tree/forest and, for each *v*∈*T*, *χ*(*v*)⊆*X* is a set of vertices assigned to the node *v*∈*T*, such that

1. each *x*∈*X* occurs in at least one *χ*(*v*);
2. for all *x*∈*X*,{*v*∣*x*∈*χ*(*v*)} induces a connected subtree of *T*;
3. for all *e*∈*E*, there is a node *v*∈*T*, such that *e*⊆*χ*(*v*).

The *treewidth* of a tree decomposition (*T*,*χ*) is defined as max*u*∈*T*|*χ*(*u*)|−1.

**Fig. 2:**
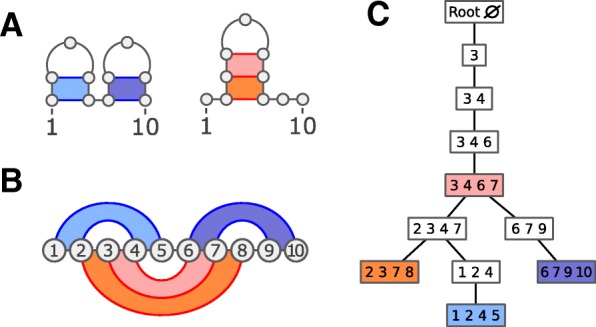
Toy example of a tree decomposition associated with two target structures in the stacking energy model (where the four positions of each base pair stack depend on each other). Two target secondary structures (**a**) are merged into a joint hypergraph (**b**), whose hyperedges correspond to the quadruplets of positions involved in base pair stacks (colored). A valid tree decomposition (**c**) for the hypergraph ensures, among other properties, that each base pair and each base pair stack is represented in at least one of its node, so that features can be correctly evaluated. The treewidth of this tree decomposition is 3, a provably optimal value for this input hypergraph

Intuitively, a tree decomposition of an (hyper)graph *G* is a tree that captures all the vertices and (hyper)edges of *G*, and properly relates dependent sub-problems to ensure consistency in a recursive computation. Figure 2 shows an optimal tree decomposition for a pair of structures under the stacking energy model.

### Fixed-parameter tractable (FPT) algorithm

Our algorithms specialize the idea of cluster tree elimination (CTE) [22], which operates on constraint networks. In this correspondence, (partial) sequences specialize (partial) assignments and the constraint network would be given by variables for each sequence position, constraints due to valid base pairing, and the set of atomic feature contributions $\mathcal {F}$.

To formalize our algorithms, which iteratively merge evaluations of partial solutions, we extend the idea of atomic feature contributions, which are evaluated at sets of the form {*x*_1_↦*v*_1_,…,*x*_*d*_↦*v*_*p*_}. Let us call the latter object a *partial sequence*. Such an object will help to specify partial knowledge on the sequence at some point of the algorithm. Easily, we can extend the definition of contributions *f* to sets {*x*_1_↦*v*_1_,…,*x*_*p*_↦*v*_*p*_}, where {*x*_1_…*x*_*p*_} is any super-set of dep(*f*) by ignoring the superfluous assignments *x*↦*v*, where *x*∉dep(*f*).

Moreover, to ensure a uniform algorithmic treatment of contributions, it is convenient to encode the weight *π* of each feature in its contributions. This transformation works by multiplying all contributions with ln(*π*), where *π* is the weight of the corresponding feature, since then *exp*(− ln(*π*)*f*(*S*))=*π*^−*f*(*S*)^.

Let us now specify the concrete set $\mathcal {F}$ of contributions that we use for the design in the stacking energy model targeting GC% and structures $\mathcal {R}$ with weights *π*_0_,…,*π*_*k*_. The set $\mathcal {F}$ thus consists of

- the transformed contributions $\ln (\pi _{0})f^{\textsf {GC}}_{i}$ for the GC*%* feature (*i*=1,…,*n*);
- the transformed contributions ln(*π*_*ℓ*_)*fij*Stack for each structure $R_{\ell }\in \mathcal {R}$ and (*i*,*j*)∈*R*_*ℓ*_.

By these definitions, the set $\mathcal {F}$ encodes the partition function $Z_{{\pi _{0},\dots,\pi _{k}}}$ of Eq. (2).

#### Partition function and stochastic backtracking

We compute the partition function (as specified by $\mathcal {F}$) by dynamic programming based on a tree decomposition of $G_{\mathcal {F}}$, the dependency graph associated with $\mathcal {F}$. Note, that analogous algorithms could be easily derived to count valid sequences, or list sequences having minimum free energy.

Our algorithms are formulated to process a *cluster tree* of $\mathcal {F}$, which is a tuple (*T*,*χ*,*ϕ*), where (*T*,*χ*) is a tree decomposition of $G_{\mathcal {F}}$, and *ϕ*(*v*) represents a set of functions *f*, each uniquely assigned to a node *v*∈*T*; dep(*f*)⊆*χ*(*v*) and $\phi (v)\cap \phi (v')=\varnothing $ for all *v*≠*v*^′^.

Two further notions are essential for our algorithms: for two nodes *v* and *u* of the cluster tree, define their *separator* as sep(*u*,*v*):=*χ*(*u*)∩*χ*(*v*); moreover, we define the *difference positions* from *u* to an adjacent *v* by diff(*u*→*v*):=*χ*(*v*)−sep(*u*,*v*).

Since our algorithms iterate over specific sets of sequence positions, we moreover define the *set*$\mathcal {P\!S}(\mathcal {Y})$*of all partial sequences determining the positions of*$\mathcal {Y}\subseteq \{1,\dots,n\}$ in all combinations of nucleotides {A,C,G,U}, i.e. for $\mathcal {Y}=\{y_{1},\dots,y_{q}\}$,

$$\begin{array}{*{20}l} \mathcal{P\!S}(\mathcal{Y}) = \{ & \{ y_{i}\mapsto v_{i} \mid i=1,\dots,q \} \\& | (v_{1},\dots,v_{q}) \in \{{\mathsf{A}},{\mathsf{C}},{\mathsf{G}},{\mathsf{U}}\}^{q} \}. \end{array} $$

We assume the following properties of the given cluster tree (reflecting $\mathcal {F}$):

- *T* is connected and contains a dedicated node *r*, with $\chi (r)=\varnothing $ and $\phi (r)=\varnothing $. If such a root does not exist, it can be added to the tree decomposition and connected to one node in each connected component of *T*;
- all edges in the tree decomposition are oriented towards this root;
- all sets diff(*u*→*v*) are singleton: for any given cluster tree, an equivalent (in term of treewidth) cluster tree can always be obtained by inserting at most $\Theta (|\mathcal {X}|)$ additional clusters.

Algorithm 1 computes the partition function by passing messages along the directed edges *u*→*v* (which point from child *u* to its parent *v*). Each message *m* has the form of a contribution, i.e. it takes a partial sequence, depends on the positions $\text {dep}(m)\subseteq \mathcal {X}$, and yields a partition function in $\mathbb {R}$. The message from *u* to *v* represents the partition functions of the subtree of *u* for all possible partial sequences in $\mathcal {P\!S}(\text {sep}({u},{v}))$. Induction over *T* lets us show the correctness of the algorithm (Additional file 1: Section H). After running Algorithm 1, multiplying the 0-ary messages sent to the root *r* yields the total partition function (i.e. due to proper encoding the partition function of our design problem) through $\prod _{({u} \rightarrow {r})\in T} {m_{{u}\rightarrow {r}}}({\varnothing })$.


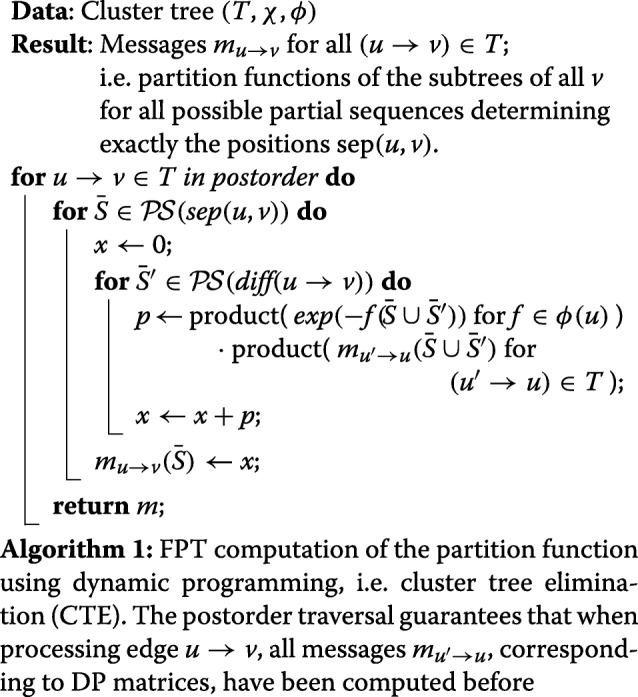


The partition functions can then direct a stochastic backtracking procedure to sample sequences from the Boltzmann distribution (according to $\mathcal {F}$). For an expanded cluster tree, after the messages *m*_*u*→*v*_ for the edges in the tree decomposition are generated by Algorithm 1, one can repeatedly call Algorithm 2, each time randomly drawing another sequence from the Boltzmann distribution.

### Complexity considerations

Let *s* denote the maximum size of any separator set sep(*u*,*v*) and *D* denote the maximum size of diff(*u*→*v*) over (*u*,*v*)∈*E*. In the absence of specific optimizations, running Algorithm 1 requires $\mathcal {O}\left ((|\mathcal {F}|+|V|)\cdot 4^{w+1}\right)$ time and $\mathcal {O}(|V|\cdot 4^{s})$ space; Algorithm 2 would require $\mathcal {O}((|\mathcal {F}|+|V|)\cdot 4^{D})$ per sample on arbitrary tree decompositions (Additional file 1: Section I). W.l.o.g. we assume that *D*=1; note that tree decompositions can generally be transformed, such that diff(*u*→*v*)≤1. Moreover, the size of $\mathcal {F}$ is linearly bounded: for *k* input structures for sequences of length *n*, the energy function is expressed by $\mathcal {O}(n\,k)$ functions. Finally, the number of cluster tree nodes is in *O*(*n*), such that $|\mathcal {F}|+|V| \in \mathcal {O}(n\,k)$.


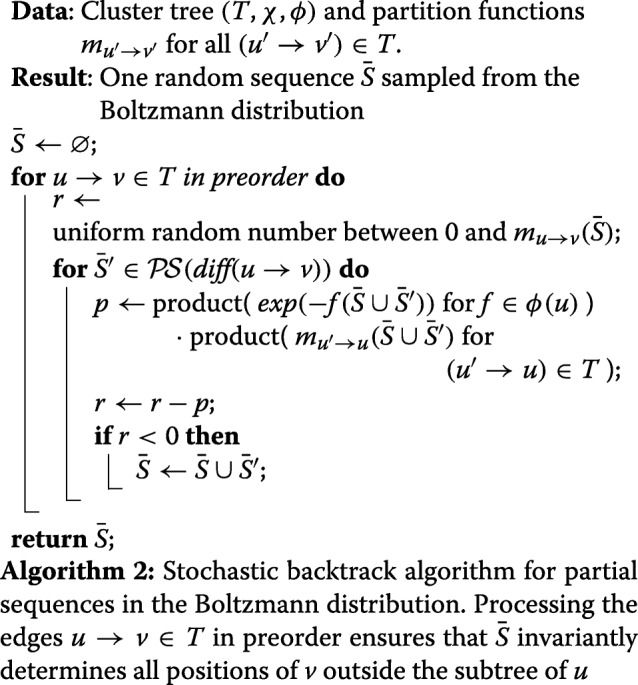


#### **Theorem 1**

(Complexities) Given are sequence length *n*, *k* target structures, and treewidth *w*. *t* sequences are generated from the Boltzmann distribution in *O*(*n* *k* 4^*w*+1^+*t* *n* *k*) time.

By this theorem, the complexity is polynomial for fixed value of *w*, and Boltzmann sampling in our setting is thus fixed parameter tractable (FPT) in the treewidth. The complexity of the precomputation can be further improved to $\mathcal {O}\left (n\,k\,2^{w+1}\,2^{c}\right)$, where *c* (*c*≤*w*+1) is the maximum number of connected components represented in a node of the tree decomposition (Additional file 1: Section J).

Note that in this complexity analysis, we do not include time and space for computing the tree decomposition itself, since we observed that the computation time of tree decomposition (GreedyFillIn, implemented in LibTW by [23]) for multi-target sampling is negligible compared to Algorithm 1 (Additional file 1: Sections B and G).

### Design within expressive energy models

In order to capture realistic energy models like the Turner model or pseudoknot models like HotKnots [24], our sampling strategy can be extended in two ways: 1) either by directly sampling based on more expressive energy models or 2) by sampling in a simple energy model which can be used to approximate sampling in more complex models. In practice, complex energy models have a strong influence on the treewidth (of optimal tree decompositions) of the dependency graph and thus on the computational complexity of our approach. Therefore, it is interesting to consider—in addition to the stacking energy model—other stripped-down variants of the nearest neighbor model, which could offer a compromise between low-complexity (as due to the stacking energy model) and the high-accuracy of the Turner model.

**Exact energy models.** A first model, which is particularly promising, is the *stacking energy model*. This model only assigns energy contributions *ΔG*(*x*_*i*_,*x*_*j*_,*x*_*i*+1_,*x*_*j*−1_) to stacks consisting of two nested base pairs (*i*,*j*) and (*i*+1,*j*−1). Within our framework, this energy model is captured by contributions *f*_*S*_({*x*_*i*_↦*s*_*i*_,*x*_*i*+1_↦*s*_*i*+1_,*x*_*j*−1_↦*s*_*j*−1_,*x*_*j*_↦*s*_*j*_}):=*ΔG*(*x*_*i*_,*x*_*j*_,*x*_*i*+1_,*x*_*j*−1_) associated with stacks occurring in at least one of the input structures.

Complex *loop-based* energy models—e.g. the Turner model which, among others, includes energy terms for special loops and dangling ends—can also be encoded exactly as instances of our general framework. Namely, each loop *L* involving positions *x*_1_,…, *x*_*p*_ will be modeled by a contribution *f*_*L*_({*x*_1_ ↦ *s*_1_,…,*x*_*p*_ ↦ *s*_*p*_}):=*ΔG*(*s*_1_,…,*s*_*p*_), where *ΔG*(*s*_1_,…,*s*_*p*_) is the energy assigned to the loop in the energy model for a given nucleotide content *s*_1_,…,*s*_*p*_. Note that the maximum arity of contributions constitutes a lower bound on the treewidth, which may impact the practical complexity of our algorithms. For instance, loop contributions in the Turner 2004 model [25] may depend on up to nine bases for interior loops, with a total of 5 unpaired bases (“2x3” interior loops)— although all other energy contributions, including dangling ends, only depend on at most four nucleotides.

**Approximating Turner Energy using Simpler Energy Models.** To capture the realistic Turner model *E*_T_ more efficiently, we exploit the tight correlation between *E*_T_ and the fitted stacking model *E*_st_ (Additional file 1: Section F). More precisely, we observed a structure-specific affine dependency between the Turner and stacking energy models, so that *E*_T_(*S*;*R*)≈*γ*·*E*_st_(*S*;*R*)+*δ* for any structure *R* and sequence *S*. We inferred the (*γ*,*δ*) parameters from a set of sequences generated with homogeneous weights *w*=*e*^*β*^, tuning only GC% to a predetermined value. Finally, we adjusted the targeted energies within our stacking model to $E_{\text {st}}^{\star } = (E_{\mathrm {T}}^{\star }- \delta)/\gamma $ in order to reach, on average, the targeted energy $E_{\mathrm {T}}^{\star }$ in the Turner model.

### Extension to multidimensional Boltzmann sampling

The flexibility of our framework allows to support the advanced sampling technique called “multidimensional Boltzmann sampling” [26], which allows to enforce (probabilistically) additional, complex properties of the samples through an additional rejection. This technique was previously used to control GC% [9, 27] and di-nucleotide content [4] of sampled RNA sequences. Here, in addition to controlling GC% (our feature *F*_0_) we use it to target the free energies $(E^{\star }_{1},\ldots,E^{\star }_{k})$ of the individual target structures (features *F*_1_,…,*F*_*m*_).

For the multidimensional Boltzmann sampling, we require the already established ability to *sample from a weighted distribution* over the set of valid sequences, where the probability of a sequence *S* is

$$\mathbb{P}(S\mid \pmb{\pi}) = \frac{\prod_{\ell=0}^{k} \pi_{i}^{-F_{i}(S)}}{Z_{\pmb{\pi}}}, $$

where *π*:=(*π*_0_⋯*π*_*k*_) is the vector of the positive real-valued *weights*, and *Z*_*π*_ is the weighted partition function.

One then needs to *learn a weights vector* *π* such that, on average, the targeted energies are achieved by a random sequences in the weighted distribution. In other words, $\mathbb {E}(F_{\ell }(S)\mid \pmb {\pi })=E^{\star }_{\ell }, \forall \ell \in [1,k]$ and, analogously, the expectation of *F*_0_(*S*) is the targeted GC content. The expected value of *F*_*ℓ*_ is always decreasing for increasing weights *π*_*ℓ*_ (see Additional file 1: Section K). More generally, computing a suitable parameter vector *π* can be restated as a convex optimization problem, and be efficiently solved using a wide array of methods [28, 29].

In practice, we use a simple heuristics which starts from an initial weight vector *π*^[0]^:=(*e*^*β*^,…,*e*^*β*^) for *β*=1/(*RT*), T= 37^∘^, and gas constant *R*. Then, at each iteration, it generates samples $\mathcal {S}$ of sequences. The expected value of an energy *F*_*ℓ*_ is estimated as $\hat \mu _{\ell }(\mathcal {S}) = \sum _{S\in \mathcal {S}}F_{\ell }(S)/|\mathcal {S}|$, and the weights are updated at the *t*-th iteration by $\pi _{\ell }^{[t+1]} = \pi _{\ell }^{[t]}\cdot \gamma ^{\hat \mu _{\ell }(\mathcal {S})-E^{\star }_{\ell }}$. In practice, the constant *γ*>1 is chosen empirically (*γ*=1.2) to achieve effective optimization. While heuristic in nature, this basic iteration was elected in our initial version of  because of its good empirical behavior.

A further *rejection step* is applied to retain only those sequences whose energy for each structure *R*_*ℓ*_ belongs to $[E^{\star }_{\ell }\cdot (1-\varepsilon),E^{\star }_{\ell }\cdot (1+\varepsilon)]$, for *ε*≥0 some predefined *tolerance*. The rejection approach is justified by the following considerations: i) *Enacting an exact control over the energies would be technically hard and costly.* Indeed, controlling the energies through dynamic programming would require explicit convolution products, generalizing [30], inducing additional *Θ*(*n*^2*k*^) time and *Θ*(*n*^*k*^) space overheads; ii) *Induced distributions are typically concentrated.* Intuitively, unless sequences are fully constrained individual energy terms are independent enough so that their sum is concentrated around its mean – the targeted energy (cf. Fig. 5). For base pair-based energy models and special base pair dependency graphs (paths, cycles…) this property rigorously follows from analytic combinatorics, see [31] and [32]. In such cases, the expected number of rejections before reaching the targeted energies remains constant when $\varepsilon \ge 1/\sqrt {n}$, and *Θ*(*n*^*k*/2^) when *ε*=0.

### #P-hardness of counting valid designs

While efficient, both in practice and in theory for graphs of bounded treewidth, our algorithms remain exponential in the worst case scenario, since the treewidth of a dependency graph can then become arbitrarily large. This exponential complexity in the worst case appears to be intrinsic. Indeed, we show that a specialization of our core problem, namely the enumeration of designs that respect canonical base pairing rules (A⇔U,G⇔C,G⇔U) is *#*P-hard, even when the dependency graph is bipartite and connected. The existence of a polynomial time algorithm for computing the partition function of Eq. 2 is thus unlikely, as it would imply that # P=FP and, in turn, that P=NP.

To establish that claim, we consider a dependency graph *G*=(*V*_1_∪*V*_2_,*E*) that is connected and bipartite (*E*∩(*V*_1_×*V*_2_)=*E*). Note that, assigning a nucleotide to a position *u*∈*V* constrains the parity ({A,G} or {C,U}) of all positions in the connected component of *u*. For this reason, we restrict our attention to the counting of valid designs *up to trivial symmetry* (A⇔C/G⇔U), by constraining the positions in *V*_1_ to A and G. Let Designs^⋆^(*G*) denote the subset of all designs for *G* under this constraint, noting that *#*Designs(*G*)=2·|Designs^⋆^(*G*)|.

Finally, let IndSets(*G*) denote the set of all independent sets in the connected graph *G*; recall that an *independent set* of *G*=(*V*,*E*) is a subset *V*^′^⊆*V* of nodes that are not connected by any edge in *E*.

#### **Proposition 1**

|Designs^⋆^(*G*)|=|IndSets(*G*)|.

#### *Proof*

Consider the mapping *Ψ*:Designs^⋆^(*G*)→IndSets(*G*),*f*↦{*v*∈*V*∣*f*(*v*)∈{A,C}}.

We show that *Ψ* is bijective:

- *Ψ* is injective, i.e. *Ψ*(*f*)≠*Ψ*(*f*^′^) for all *f*≠*f*^′^. If *f*≠*f*^′^, then there exists a node *v*∈*V* such that *f*(*v*)≠*f*^′^(*v*). We discuss only the case *v*∈*V*_1_, where we restricted the nucleotides to A and G. Then, {*f*(*v*),*f*^′^(*v*)} must equal {A,G}, such that either *v*∈*Ψ*(*f*) or *v*∈*Ψ*(*f*^′^).
- *Ψ* is surjective, i.e. there is a preimage for each element *I*∈IndSets(*G*). Define *f*∈Designs^⋆^(*G*) as
  $$ f(v) = \left\{\begin{array}{ll} {\mathsf{A}}& \text{if}\ v\in V_{1}\ \text{and}\ v\in I \\ {\mathsf{C}}& \text{if}\ v\in V_{2}\ \text{and}\ v\in I \\ {\mathsf{G}}& \text{if}\ v\in V_{1}\ \text{and}\ v\not\in I\\ {\mathsf{U}}& \text{if}\ v\in V_{2} \text{and}\ v\not\in I \end{array}\right.  $$
  One easily verifies that *Ψ*(*f*)=*I*. It remains to show that *f* is a valid design for *G*, i.e. for each (*v*,*v*^′^)∈*E*, $\{f(v),f(v')\}\in \mathcal {B}$; please recall that we defined $\mathcal {B}$ as the set of all valid nucleotide pairs. Assume there is an edge (*v*_1_,*v*_2_)∈*E*, violating $\{f(v_{1}),f(v_{2})\} \in \mathcal {B}$. Since *G* is bipartite, *v*_1_∈*V*_1_ and *v*_2_∈*V*_2_, such that *f*(*v*_1_)∈{A,G} and *f*(*v*_2_)∈{C,U}. This implies that among all possible {*f*(*v*_1_),*f*(*v*_2_)} only {A,C} is not in $\mathcal {B}$, which in turn requires *v*_1_∈*I* and *v*_2_∈*I*. Therefore, since *I* is an independent set, the edge (*v*_1_,*v*_2_)∈*E* cannot exist. ■

□

Counting independent sets in bipartite graphs (*#*BIS) is a well-studied problem, shown to be #P-hard [33] even on connected graphs. Now assume the existence of an efficient (polynomial-time) algorithm $\mathcal {A}$ for computing |*#*Designs(*G*)| on connected (bipartite) graphs. Then, running $\mathcal {A}$ and returning |*#*Designs(*G*)|/2 constitutes an efficient algorithm for *#*BIS on connected graphs. In other words, any efficient algorithm for #Designs implies an efficient algorithm for *#*BIS, thus our conclusion that #Designs is *#*P-hard.

Proposition 1 also strongly impacts the complexity of computing the partition function. Indeed it implies that, among the 4^*k*^ possible assignments of nucleotides to *k* connected positions (in the base-pair dependency graph), at most 2^*k*^ are compatible with base pairing rules. One can thus sharply reduce the complexity of Algorithm 1 by restricting the precomputations to compatible assignments.

For a discussion on the implications of our hardness results beyond exact counting see Additional file 1: Section L.

## Results

We implemented the core algorithms in C++, resulting in the tool , available at: https://github.com/yannponty/RNARedPrint.

 takes a set of target structures, as well as weights for each energy feature and GC%, and generates a sample set of sequences compatible with the structures in the corresponding Boltzmann distribution; it currently supports the stacking energy model and the base pair energy model (Additional file 1: Section F).

Moreover, we provide two Python wrapper scripts. The first script targets prescribed energies using multi-dimensional Boltzmann sampling. For a given set of secondary structures, together with prescribed target energies and target GC%, this script generates a series of sequences that satisfy the target values for the energy and GC% features within configurable tolerances. Notably, these target energies are actual free energies in the realistic Turner energy model, and are targeted by efficiently sampling in the stacking energy model, and filtering sequences based on the RNAeval tool from the Vienna package [34]. The second script generates high quality seed sequences suitable for negative RNA design. The details of this approach are described in the subsections below.

### Practical efficacy of Boltzmann sampling for sequences

**Fig. 3:**
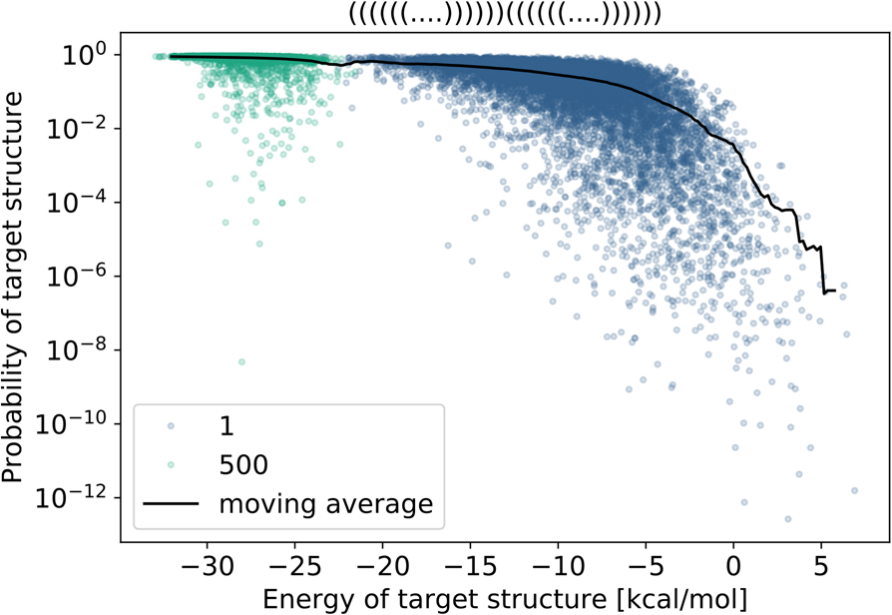
Comparison of free energy and Boltzmann probability for 10 000 uniform (*π*=1; blue dots) and Boltzmann distributed (*π*=500; green dots) sequences, targeting a simple structure consisting of two adjacent helices

First, we show how seed sequences can be generated in a Boltzmann distribution, leading to designs that are substantially more stable that those generated uniformly. As can be seen in Fig. 3 and Additional file 1: Section A, sequences generated in the Boltzmann distribution not only reach lower free-energies than those generated in a uniform setting, but also achieve better Boltzmann probabilities. While the former is expected since the Boltzmann distribution explicitly favors low-energy candidates, the latter is somewhat surprising, since the Boltzmann probability of a target structure could, in principle, decrease under Boltzmann sampling due to the partition function growing faster than the Boltzmann factor. The empirical superiority of Boltzmann sampling appears robust to the target structure length and topology, as demonstrated by prior work [9].

**Fig. 4:**
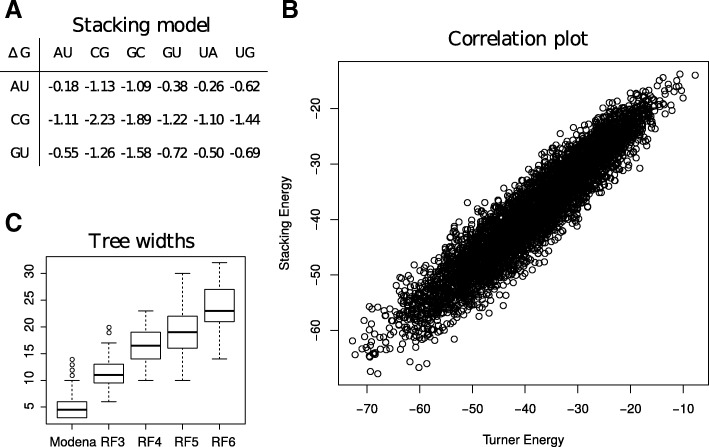
A fitted energy model based on stacking pairs (**a**) leads to approximated free-energies that are highly correlated (R=0.99) with free-energies in the Turner energy model (**b**), yet induces tree widths that are amenable to practical sampling (**c**)

However, while in principle feasible, sampling in a Boltzmann distribution directly using the Turner energy model may induce extreme computational demands, with treewidths scaling at least as large as the number of nucleotides in the largest loop. Fortunately, we found that intricacy of the Turner energy model can be circumvented with minimal loss of precision by using a simpler stacking energy model. As shown in Fig. 4, a simple stacking energy model, whose design principles are further described in Additional file 1: Section F, can be used to approximate the Turner energy model very adequately (correlation coefficient *R*=0.99) in the context of sequence design. Using this simpler model greatly reduces the treewidth, and thus the computational requirements of the whole method even for complex instances.

### Effectively targeting Turner energies using multi-dimensional sampling

We used our Boltzmann sampling strategy (Algorithms 1 and 2), to sample valid sequences for given target structures and weights *π*_1_,…,*π*_*k*_. Moreover, we used multi-dimensional Boltzmann sampling to target specific energies and GC%. Our tool  evaluates energies according to the stacking energy model *E*_st_, whose parameters were fitted to best approximate Turner energies. As well, we implemented and fitted a base pair energy model for , which was not studied for its targeting performance (both models: Additional file 1: Section F).

**Fig. 5:**
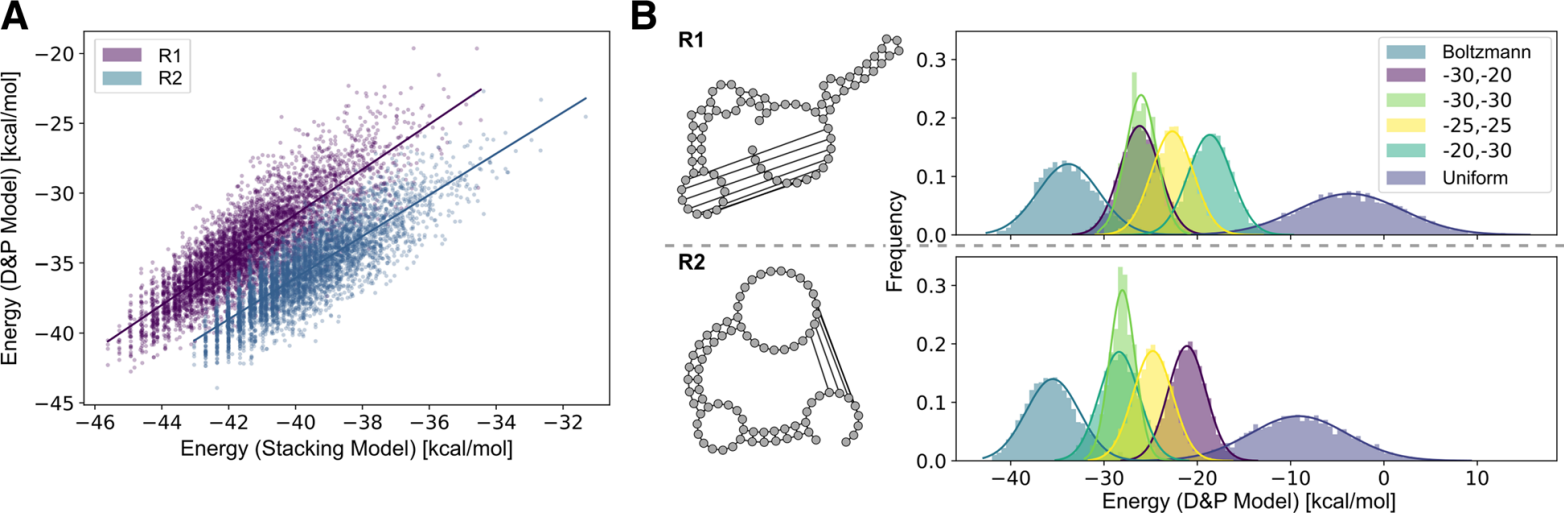
Targeting specific energies for pseudoknotted structures using multi-dimensional Boltzmann sampling. **a** Linear fits between the energies in the stacking model to the realistic pseudoknot energy model by Dirks and Pierce (D&P) for initially sampled sequences and both target structures R1 and R2 (shown in **b**). The good match (respective *R*^2^ values of 0.846 and 0.841) enables more efficient targeting of Turner energies based on targeting stacking model energies. **b** Resulting D&P energy distributions for the two target structures R1 and R2 when aiming for the respective free energies − 30 and − 20,−30 and −30,−25 and −25,−20 and −30 kcal/mol. These demonstrate the effectivity of our adaptive multi-dimensional Boltzmann sampling procedure, especially by comparing the distributions to those of uniform and Boltzmann sampled sequences, with homogeneous weights 1 and *e*^*β*^, respectively

Figure 5 illustrates how well complex realistic energy models can be approximated based on simpler, but better tractable ones. For the two target structures of Fig. 5a, b shows the good fit between realistic energies in the full-fledged Dirks and Pierce energy model for pseudoknots (D&P model) and energies in the stacking energy model, which is obtained for each of the two target structures (with respective *R*^2^ values of 0.846 and 0.841). For the shown fits we sampled *n*=10 000 sequences, targeting a GC% of 60%. For an example instance of the Modena benchmark with two pseudoknotted target structures, Fig. 5b shows the Turner energy distributions of the single structures as they result from sampling with different weight parameters. The figure illustrates how our multidimensional Boltzmann sampling strategy can, to a large extent, independently shift the Turner energies of sampled sequences towards prescribed targets. See Additional file 1: Section D for a further example with three pseudoknot-free target structures.

### Generating high-quality seeds for further optimization

We empirically evaluated  for generating seed sequences targeting multiple (pseudoknotted) structures, possibly followed by subsequent local optimizations. As a baseline for comparison, we considered  [18], the current leading tool for multiple design. As a quality measure, we applied the objective function introduced by [18] based on [16, 35] for multi-stable design, defined as:

3 $$\begin{array}{*{20}l}  \text{MultiDefect}(S) &= \frac{1}{m} \sum_{\ell=1}^{m} (E(S, R_{\ell}) - \text{G}(S))\\ &\;\;\; \!+\frac{1}{2{{m}\choose{2}}} \sum\limits_{1\leq\ell< j\leq m}|E(S,R_{\ell}) - E(S,R_{j})|, \end{array} $$

where the free energies *E*(*S*,*R*) as well as the *ensemble free energy* G(*S*) of *S* are computed by RNAfold [34] in the pseudoknot-free case; for pseudoknotted targets, G(*S*) is approximated by the *minimum free energy* of *S* as estimated by HotKnots [24] in the energy model of [36]. Intuitively, the first term of MultiDefect captures the distance of the targets from the ensemble free energy, while the second term penalizes the dispersion of targets; MultiDefect is best (minimized) when all targets simultaneously achieve the minimum free energy of the sequence.

We considered a benchmark of six sets of target structures described in [17]: 2str, 3str, and 4str consist of non-pseudoknotted structures, while PK60, PK80, and LE80 contain pseudoknotted structures. Based on , we generated at least 1 000 seed sequences with similar energies for all target structures, for each instance of the benchmark. For this purpose, we determine good common target energies that can be successfully targeted for all single target structures simultaneously. Generally, we targeted 60% GC%. Additional file 1: Section E provides detailed results from this iterative procedure, which works similarly to the previously described multi-dimensional Boltzmann sampling. In particular, we observe that most of the benchmark inputs finish within only few iterations, where each iteration requires little time (confer Additional file 1: Section C).

We compared the MultiDefect value of the derived sequences against that of seed sequences, uniformly sampled using . Moreover, for both sets we used an adaptive greedy walk [18] to *minimize* the MultiDefect function. At each step, the local search re-samples (uniformly at random) the positions of a randomly selected component in the base pair dependency graph, accepting the modification only if it results in a gain. We performed 500 greedy descent steps in the case of pseudoknot-free data-sets 2str, 3str, and 4str; and 200 steps for the pseudoknotted ones PK60, PK80, and LE80.

**Fig. 6:**
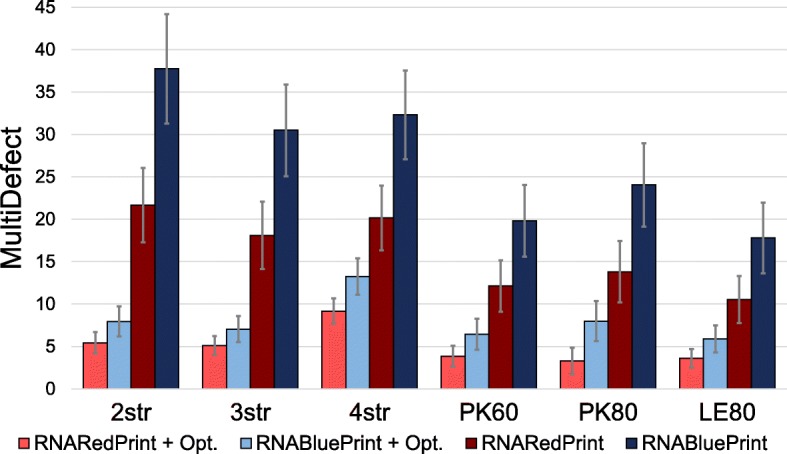
Comparison of the MultiDefect (see Eq. (3); smaller values → better designs) for sequences sampled by  and uniform sampling  for six benchmark sets. For both sampling schemes, we show the MultiDefects of the sampled sequences, as well as results after further optimization by local search (“+ Opt.”)

The results, shown in Fig. 6, reveal that Boltzmann-sampled sequences outperform uniform seeds on every data-set, leading to average improvements in MultiDefect values ranging from 7.26 (LE80) to 16.05 (2str) units. Remarkably, this improvement is observed for both terms in MultiDefect (see Additional file 1: Section M). This means that  produces sequences whose targets are substantially closer to the ensemble free energy *and* have more similar stability across targets. In fact, for every sequence in our benchmark, consisting of 332 sets of target structures, we observed better MultiDefect for Boltzmann sampling than for uniform sampling (see Additional file 1: Section N). Notably,  performs equally well in the presence of pseudoknots; the difficulty rather lies in the computation of the MultiDefect function, since free energy minimization is costly in the presence of pseudoknots [37] and good implementations are scarce.

Moreover, for all instances as well, the Boltzmann designs remain superior even after local optimizations, as shown by todo 6 and Additional file 1: Section M and N. This observation is consistent with a superior quality of the starting point for the greedy walk, probably leading to better local minima of the MultiDefect function. However, it should be noted that the greedy walk is based on the uniform sampling of , and thus can be expected to partially level the advantages of Boltzmann sampling. In future work, we hope to improve this aspect by exploiting Boltzmann sampling during the optimization run.

## Conclusion

Based on a general framework and efficient algorithms, we introduce a novel approach to design RNA sequences while targeting very specific complex properties. In particular, we describe the targeting of the free energies of multiple target structures and the GC-content. Our method combines a fixed-parameter tractable (FPT) sampling algorithm with multi-dimensional Boltzmann sampling over distributions controlled by expressive RNA energy models. We demonstrate that the approach, despite of its theoretical hardness, performs well on typical multi-stable RNA design instances in practice. This good performance is a direct consequence of the approximability of Turner energies as well as the systematic algorithmic framework. By conducting a proof-of-concept study on an established benchmark set for negative multi-target RNA design (including pseudoknotted instances), we showcase a typical application of our tool . In this study, the approach generates significantly better seed sequences than the previously best available method (uniform sampling). Our results strongly suggest that the presented technique for positive design can be highly beneficial in future negative design approaches. To substantiate our work additionally, we establish the *#*P-hardness of uniform sampling which, from a complexity-theoretic point of view, motivates the FPT, tree decomposition-based nature of our method.

In this way, our framework enables new possibilities in the field of RNA sequence design. As particular advantage over previous sequence generation methods, it is extensible to include various more complex sequence constraints, including mandatory/forbidden motifs at specific positions or anywhere in the designed sequences, by adapting formal language constructs of Zhou et al. [10]. In future work, negative design principles could be explicitly supported at the generation stage, for instance by penalizing a set of alternative helices/structures. We moreover envision using positive design to assess the significance of observed properties. Critically, our current perception of statistical significance suffers from overly simplistic simple null models (e.g. dinucleotide shuffling) used to model random RNAs [38]). Here, our approach promises fundamental improvements by constructing null models of random sequences that satisfy multiple complex constraints.

## Additional file

Additional file 1 The Supplemental Material contains additional information on methods and parameters; elaboration of theory and proofs; as well as additional and detailed empirical results. (PDF 6642 kb)

## Abbreviations

#P: Complexity class “Sharp P”
DP: Dynamic programming
D&P model: Pseudoknot energy model by Dirks and Pierce
FPT: Fixed-parameter tractable
GC%: GC-content

## References

[1] Kushwaha M, Rostain W, Prakash S, Duncan JN, Jaramillo A (2016). Using RNA as molecular code for programming cellular function. ACS Synth Biol.

[2] Rodrigo G, Jaramillo A (2014). RiboMaker: computational design of conformation-based riboregulation. Bioinformatics.

[3] McCaskill JS (1990). The equilibrium partition function and base pair binding probabilities for RNA secondary structure. Biopolymers.

[4] Zhang Y, Ponty Y, Blanchette M, Lécuyer E, Waldispühl J (2013). SPARCS: a web server to analyze (un)structured regions in coding RNA sequences. Nucleic Acids Res.

[5] Wachsmuth M, Findeiß S, Weissheimer N, Stadler PF, Mörl M (2013). De novo design of a synthetic riboswitch that regulates transcription termination. Nucleic Acids Res.

[6] Domin G, Findeiß S, Wachsmuth M, Will S, Stadler PF, Mörl M (2017). Applicability of a computational design approach for synthetic riboswitches. Nucleic Acids Res.

[7] Findeiß Sven, Etzel Maja, Will Sebastian, Mörl Mario, Stadler Peter (2017). Design of Artificial Riboswitches as Biosensors. Sensors.

[8] Zhu JYA, Steif A, Proctor JR, Meyer IM (2013). Transient RNA structure features are evolutionarily conserved and can be computationally predicted. Nucleic Acids Res.

[9] Reinharz V, Ponty Y, Waldispühl J (2013). A weighted sampling algorithm for the design of RNA sequences with targeted secondary structure and nucleotide distribution. Bioinformatics (Oxford, England).

[10] Zhou Y, Ponty Y, Vialette S, Waldispühl J, Zhang Y, Denise A. Flexible RNA design under structure and sequence constraints using formal languages In: Gao J., editor. ACM Conference on Bioinformatics, Computational Biology and Biomedical Informatics. ACM-BCB 2013, Washington, DC, USA, September 22-25, 2013. ACM: 2013. p. 229. 10.1145/2506583.2506623.

[11] Levin A, Lis M, Ponty Y, O’Donnell CW, Devadas S, Berger B, Waldispühl J (2012). A global sampling approach to designing and reengineering RNA secondary structures. Nucleic Acids Res.

[12] Busch A, Backofen R (2006). INFO-RNA–a fast approach to inverse RNA folding. Bioinformatics (Oxford, England).

[13] Hofacker IL, Fontana W, Stadler PF, Bonhoeffer LS, Tacker M, Schuster P (1994). Fast folding and comparison of RNA secondary structures. Monatshefte für Chemie/Chemical Monthly.

[14] Kleinkauf R, Houwaart T, Backofen R, Mann M (2015). antarna – multi-objective inverse folding of pseudoknot rna using ant-colony optimization. BMC Bioinformatics.

[15] Lyngsø RB, Anderson JWJ, Sizikova E, Badugu A, Hyland T, Hein J (2012). Frnakenstein: multiple target inverse RNA folding. BMC Bioinformatics.

[16] Höner zu Siederdissen C, Hammer S, Abfalter I, Hofacker IL, Flamm C, Stadler PF (2013). Computational design of RNAs with complex energy landscapes. Biopolymers.

[17] Taneda A (2015). Multi-objective optimization for RNA design with multiple target secondary structures. BMC Bioinformatics.

[18] Hammer S, Tschiatschek B, Flamm C, Hofacker IL, Findeiß S (2017). RNAblueprint: flexible multiple target nucleic acid sequence design. Bioinformatics (Oxford, England).

[19] Ding Y, Lawrence CE (2003). A statistical sampling algorithm for RNA secondary structure prediction. Nucleic Acids Res.

[20] Bulatov AA, Dyer M, Goldberg LA, Jerrum M, Mcquillan C (2013). The expressibility of functions on the boolean domain, with applications to counting CSPs. J ACM.

[21] Cai J-Y, Galanis A, Goldberg LA, Guo H, Jerrum M, Štefankovič D, Vigoda E (2016). # BIS-hardness for 2-spin systems on bipartite bounded degree graphs in the tree non-uniqueness region. J Comput Syst Sci.

[22] Dechter Rina (2013). Reasoning with Probabilistic and Deterministic Graphical Models: Exact Algorithms. Synthesis Lectures on Artificial Intelligence and Machine Learning.

[23] van Dijk T, van den Heuvel J-P, Slob W. Computing treewidth with LibTW. Technical report, University of Utrecht. 2006. http://treewidth.com/treewidth/docs/LibTW.pdf.

[24] Ren J, Rastegari B, Condon A, Hoos HH (2005). Hotknots: heuristic prediction of RNA secondary structures including pseudoknots. RNA (New York, N.Y.).

[25] Turner DH, Mathews DH (2009). NNDB: the nearest neighbor parameter database for predicting stability of nucleic acid secondary structure. Nucleic Acids Res.

[26] Bodini O, Ponty Y. Multi-dimensional Boltzmann sampling of languages. In: Proceedings of the 21st International Meeting on Probabilistic, Combinatorial, and Asymptotic Methods in the Analysis of Algorithms (AofA’10). DMTCS: 2010. p. 49–64.

[27] Waldispühl J, Ponty Y (2011). An unbiased adaptive sampling algorithm for the exploration of RNA mutational landscapes under evolutionary pressure. J Comput Biol J Comput Mol Cell Biol.

[28] Denise A, Ponty Y, Termier M (2010). Controlled non-uniform random generation of decomposable structures. Theor Comput Sci.

[29] Bendkowski M, Bodini O, Dovgal S. Polynomial tuning of multiparametric combinatorial samplers. 2017. arXiv preprint arXiv:1708.01212.

[30] Cupal J, Hofacker IL, Stadler PF. Dynamic programming algorithm for the density of states of RNA secondary structures In: Hofstaedt R, Lengauer T, Loeffler M, Schomburg D, editors. German Conference on Bioinformatics. Leipzig: 1996. p. 184–6.

[31] Bender EA, Richmond LB, Williamson S (1983). Central and local limit theorems applied to asymptotic enumeration. iii. matrix recursions. J Comb Theory Ser A.

[32] Drmota M (1997). Systems of functional equations. Random Struct Algoritm.

[33] Ge Q, Štefankovič D (2012). A graph polynomial for independent sets of bipartite graphs. Comb Probab Comput.

[34] Lorenz R, Bernhart SH, zu Siederdissen CH, Tafer H, Flamm C, Stadler PF, Hofacker IL (2011). ViennaRNA Package 2.0. Algorithms Mol Biol.

[35] Flamm C, Hofacker IL, Maurer-Stroh S, Stadler PF, Zehl M (2001). Design of multistable RNA molecules. RNA (New York, N.Y.).

[36] Dirks RM, Pierce NA (2003). A partition function algorithm for nucleic acid secondary structure including pseudoknots. J Comput Chem.

[37] Sheikh Saad, Backofen Rolf, Ponty Yann (2012). Impact of the Energy Model on the Complexity of RNA Folding with Pseudoknots. Combinatorial Pattern Matching.

[38] Rivas E, Clements J, Eddy SR (2017). A statistical test for conserved rna structure shows lack of evidence for structure in lncrnas. Nat Methods.

