# Supplementary material for: Fixed-parameter tractable sampling for RNA design with multiple target structures
Source: BMC Bioinformatics. 2019 Apr 25;20:209. doi: 10.1186/s12859-019-2784-7 (PMC6482512; doi:10.1186/s12859-019-2784-7)
Supplement: Supplementary file 1 — The Supplemental Material contains additional information on methods and parameters; elaboration of theory and proofs; as well as additional and detailed empirical results. (PDF 6642 kb) [file 12859_2019_2784_MOESM1_ESM.pdf]

Supplementary Material

Fixed-Parameter Tractable Sampling for RNA Design with Multiple Target Structures

Stefan Hammer, Wei Wang, Sebastian Will and Yann Ponty

A Immediate benefits of positive design for negative design

Already previous work on RNA Design, e.g. INFO-RNA and Incarnation, observed that—put briefly—negative design benefits from positive design. Here, more concretely, we simply *demonstrate* this effect (for small examples), where we consider *negative design* as designing RNA sequences that fold with high probability $Pr(R|S)$ into the given target structure R; and *positive design*, as generating one or many sequences that have a low energy for R.

For each of two small examples, a single-stem and a double-stem structure, we produce a uniform and a weighted Boltzmann sampling, each of 10 000 sequences. For each sampled sequence S, we determine the Turner energy $E(S,R)$ as well as the probability $Pr(R|S)$ of the structure R in the ensemble of sequence S (where the energies are calculated in the Turner model using RNAfold).

Note that low energy $E(S|R)$ is (non-linearly) correlated with high probability $Pr(S|R)$, the probability to see S in the Boltzmann distribution of sequences folding into R. Recall that the probability $Pr(R|S)$ can be calculated as $\exp(-E(S,R)/RT)/Z(S)$, where $Z(S) = \sum_{R'} \exp(-E(S,R')/RT)$. Consequently, Figure 1 ultimately illustrates the dependencies between $Pr(S|R)$ and $Pr(R|S)$ for uniform samples and low energy samples (where those samples can be efficiently generated due to our Boltzmann sampling strategy). For our examples, we observe (Fig. 1) that Boltzmann sampling with weight 500 (green), generates good negative designs with substantially higher probability than uniform sampling (blue).

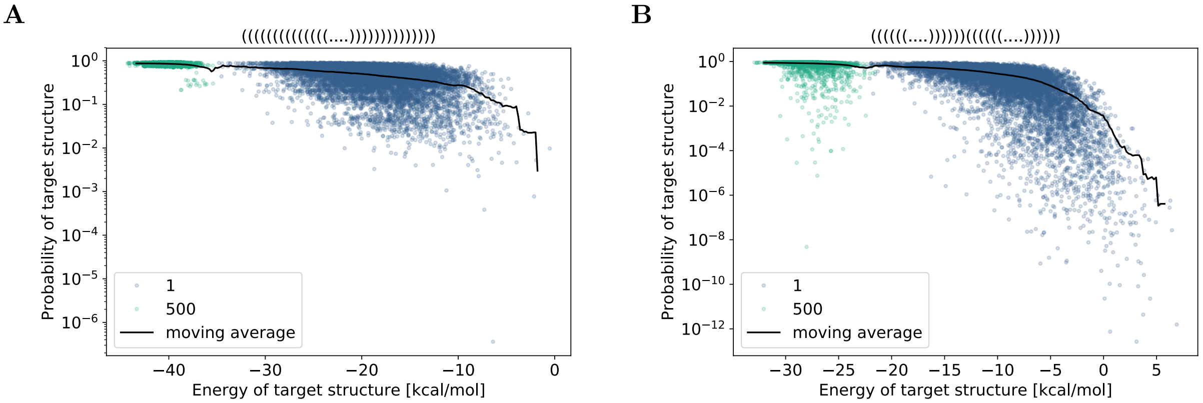

Figure 1: Comparison of uniform sampling and Boltzmann sampling with weight $\pi = 500$. We show results for two different small structures: (A) single stem structure (B) double stem structure. For each respective structure R, we plot the energies $E(S,R)$ of 10 000 sampled sequences against the probabilities $Pr(R|S)$ (in log scale). For illustrating the trend, we show moving averages over windows of ± 1 kcal. Note that while only the energy $E(S,R)$ is directly controlled by Boltzmann sampling, this strongly impacts the common negative design objective $Pr(R|S)$.

B Tree decomposition for RNA design instances in practice

For studying the typically expected treewidths and tree decomposition run times in multi-target design instances, we consider five sets of multi-target RNA design instances of different complexity. Our first set

consists of the Modena benchmark instances.

In addition, we generated four sets of instances of increasing complexity. The instances of the sets RF3, RF4, RF5, and RF6, each respectively specify 3,4,5, and 6 target structures for sequence length 100. For each instance (100 instances per set), we generated a set of k ($k = 3, \ldots, 6$) compatible structures as follows

- Generate a random sequence of length 100;
- Compute its minimum free energy structure (ViennaRNA package);
- Add the new structure to the instances if the resulting base pair dependency graph is bipartite;
- Repeat until k structures are collected.

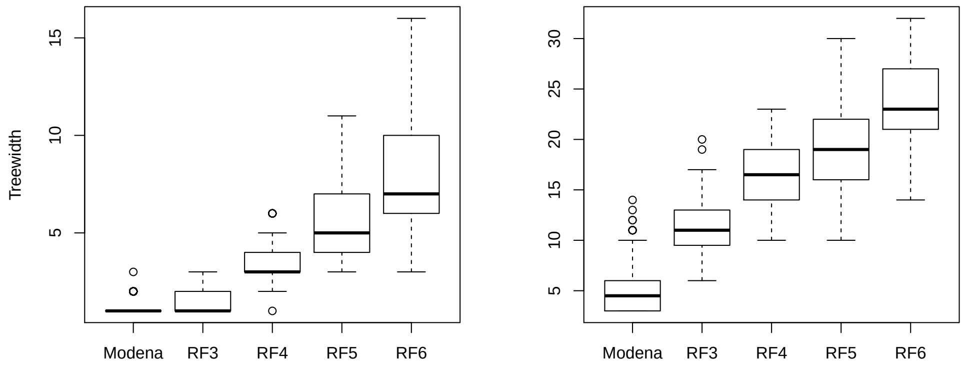

Figure 2: Treewidths for multi-target RNA design instances of different complexity. Distributions of treewidths shown as boxplots for the base pair (left) and stacking model (right).

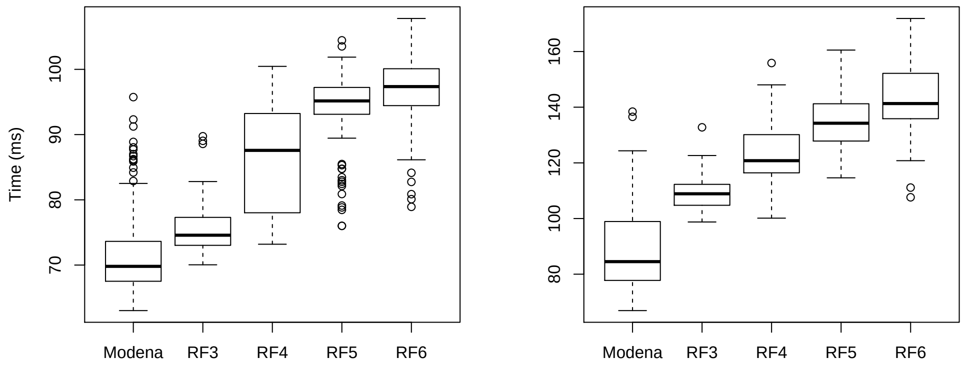

Figure 3: Computation time of tree decompositions for multi-target RNA design instances of different complexity. Distributions of times (in ms/instance) shown as boxplots for the base pair (left) and stacking model (right).

For each instance, we generated the dependency graphs in the base pair model and in the stacking model. Then, we performed tree decomposition (using strategy "GreedyFillIn" of LibTW [1]) on each dependency graph. The obtained treewidths are reported in Fig. 2, while Fig. 3 shows the corresponding run-times of the tree decomposition.

```
((((.((....)).)))).((.(((.((((.....(((..((((((.((..(((.(.....).)))..)).)).))))..)))..)))).))).))....
..(((((.....(((.(((((((.....))))..))).))).....)))))..((((((((((...))).)....))))))...((((((....))))))
......(((((.....(((...(((.((.((.(((....((......))...))).)).)))))..))).............))))).((((...)))).
```

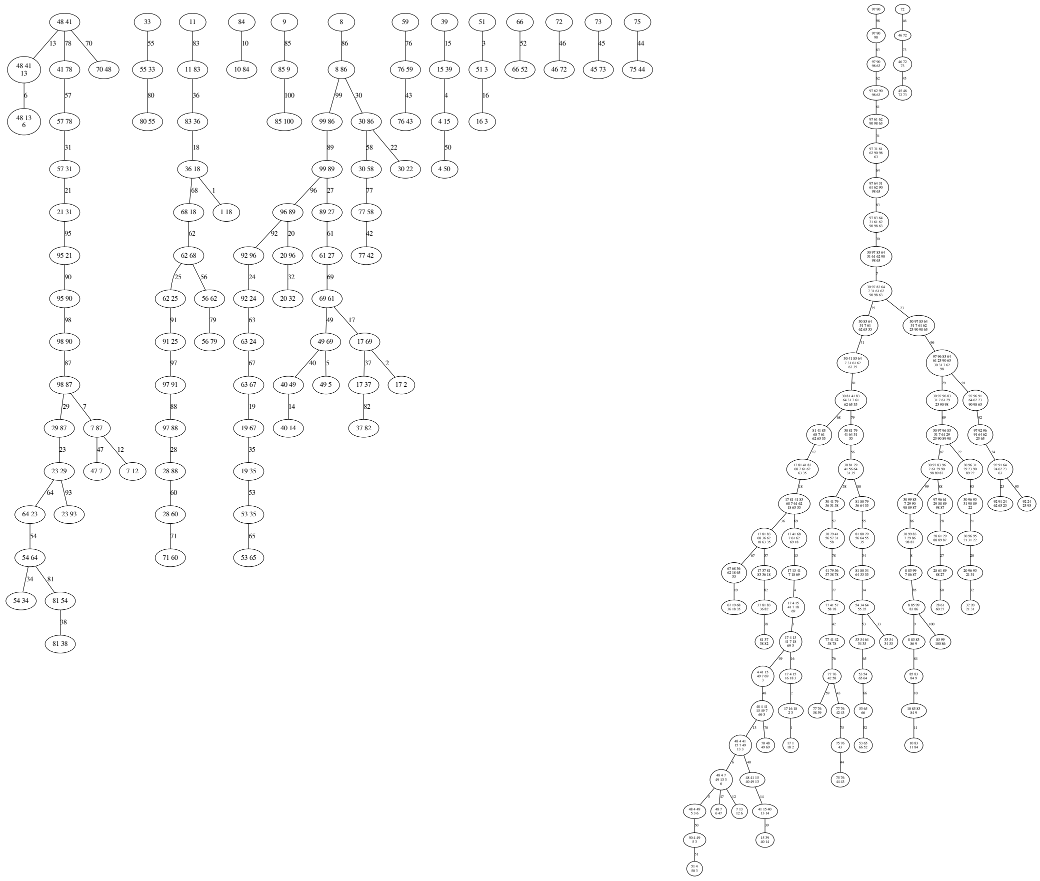

Figure 4: Example instance from RF3 (top) with its tree decompositions in the base pair (left) and stacking model (right). The respective treewidths are 2 and 12.

Finally, for further illustration, we show concrete tree decompositions for a selected instance in the benchmark set RF3 in Figure 4. This figure is provided solely for illustrational purposes: First, it depicts the tree decomposition for a typical, realistic sampling task. Second, it exemplarily illustrates the typical impact of the energy model on the tree decomposition. Compared to the tree decomposition based on the stacking model, the tree decomposition for the base pair model is much simpler. This shows in many connected components, which correspond to independently solvable sub-problems, and fewer positions per bag (i.e. smaller treewidth), which allows far more efficient evaluation per tree node. Note that the complexity of the tree decomposition ultimately shows the complexity of the dependencies that need to be considered for simultaneous energy evaluation for all target structures. This complexity is substantially increased in the stacking model over the simpler base pair model.

C Run times for Boltzmann sampling (at fixed weights)

To illustrate the efficiency and practical feasibility of our Boltzmann sampling approach, we show the distributions of run times (Intel(R) Core(TM) i7-4770 CPU) for generating 10000 samples (over the Modena benchmark instances and grouped by benchmark sub-set). Since producing a controlled Boltzmann sampling requires significant precomputation, it is informative to separately show construction and sampling times (Figure 5). Moreover, RNARedPrint can be used with a simpler base pair model or a rather complex stacking energy model, which introduces much stronger dependencies than required for uniform sampling. Technically, these stronger dependencies increase the treewidth and thus the run-time, as noticeable in Figure 5.

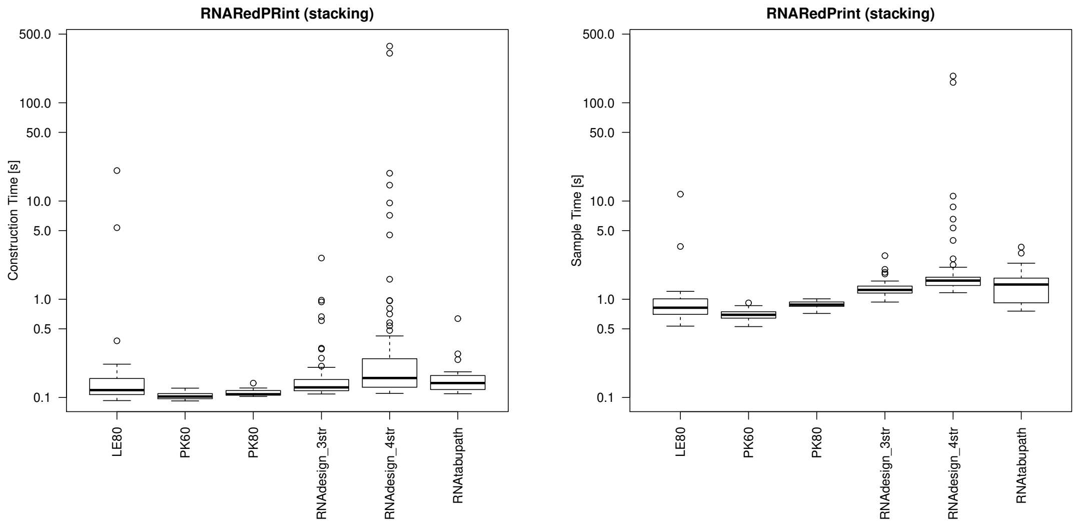

Figure 5: Boxplots showing the run-time distribution of sequence sampling with RNARedPrint for the various benchmark sets. The times are split into construction time (left) and sampling time (right). Construction includes all the precomputation, while the latter is the time needed to generate 10000 sequences.

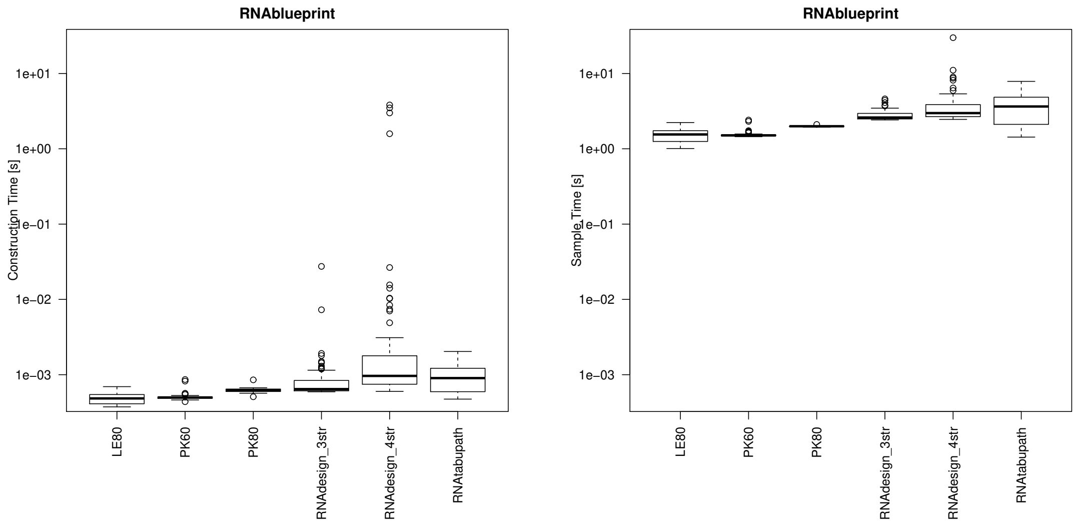

Figure 6: Boxplots showing the run-time distribution of sequence sampling with RNABluePrint for the various benchmark sets. The times are split into construction time (left) and sampling time (right). Construction includes all the precomputation, while the latter is the time needed to generate 10000 sequences.

A fair comparison to sampling times by other tools can hardly be given, since the other tools produce samplings of very different nature. In particular, Modena does not make any guarantees on properties of the sampling. Non-surprisingly, this allows it to sample significantly faster (several orders of magnitudes). Nevertheless, the comparison to run times of `RNABluePrint` is still interesting, since this tool generates—similarly to our tool—*controlled* samples. As we argue that the Boltzmann sampling is fundamentally more flexible than uniform sampling as generated by `RNABluePrint`, `RNARedPrint` performs even particularly well, beating `RNABluePrint` most of the time with only a few exceptions for particularly hard instances. The results for `RNABluePrint` are shown in Figure 6.

D Illustrating multi-dimensional Boltzmann sampling for three pseudoknot-free target structures

In analogy to Fig. 5 of the main text, Fig. 7 illustrates our multi-dimensional sampling strategy for three pseudoknot-free target structures (example instance from the `Modena` benchmark). The figure shows Turner energy distributions of single structures from sampling with different weight parameters, where weights are trained to target specific energies. For comparison, the figure shows distributions from uniform and Boltzmann sampling at respective homogeneous weights 1 and e^{β}.

Fig. 8 shows the distributions of the GC content of the samples, while targeting different combinations of energies in this experiment. For completeness, we also report corresponding results for the analogous experiment from the main text. We observe that in addition to the target energies, also the feature GC% is narrowly defined by the multi-dimensional Boltzmann sampling strategy. In comparison, for uniform sampling and Boltzmann sampling at high weight e^{β}, where the GC% is not controlled, we obtain much broader distributions.

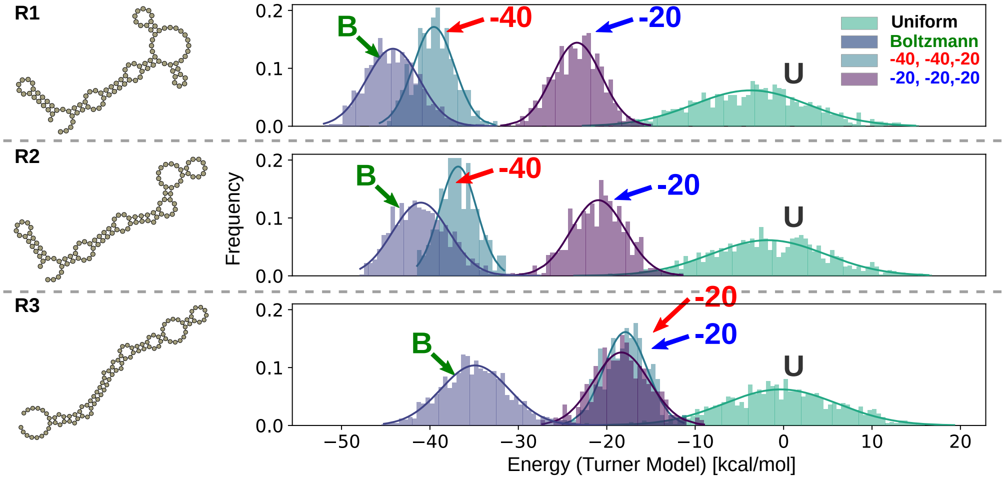

Figure 7: Targeting specific energies using multi-dimensional Boltzmann sampling. We show the Turner energy distributions for three target structures R1, R2 and R3. By targeting the respective free energies (−40, −40 and −20 kcal/mol and −20,−20 and −20 kcal/mol) for the target structures (annotated in red and blue, respectively), we demonstrate the effectivity of our adaptive multi-dimensional Boltzmann sampling procedure. Moreover, for comparison, the distributions for uniform (U) and Boltzmann (B) samples—respectively associated with homogeneous weights 1 and e^{β}.

E Effectivity of the seed sequence generation

Figure 9 shows relative numbers of finished instances and admissible sequences at each iteration of the seed generation procedure for the benchmark set `3str`. We obtain qualitatively equivalent results for all other

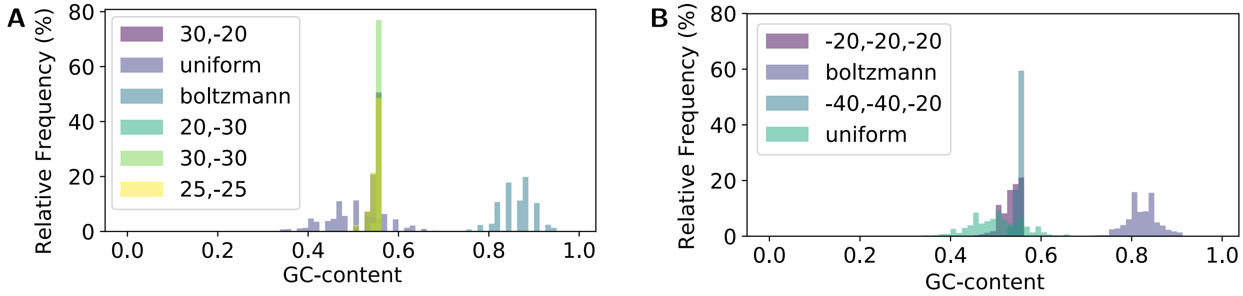

Figure 8: Distribution of the GC-content GC% in the experiments of (A) Fig. 5 of the main text and (B) Suppl. Fig. 7.

benchmark sets (not shown). The first plot illustrates the precomputation step of our strategy, where weights are calibrated for GC% of 0.6. In this step, we produce sequence of the targeted GC% to determine the linear transformation from the sampling energy model (stacking model) to the Turner model. The second plot shows the actual generation of the seed sequences after precomputation. The plot shows that most of the instances are finished after the first iterations, with only a few harder instances that require 2–3 times more iterations. Recall that the seed sequences were designed to have similar energy for all target structures.

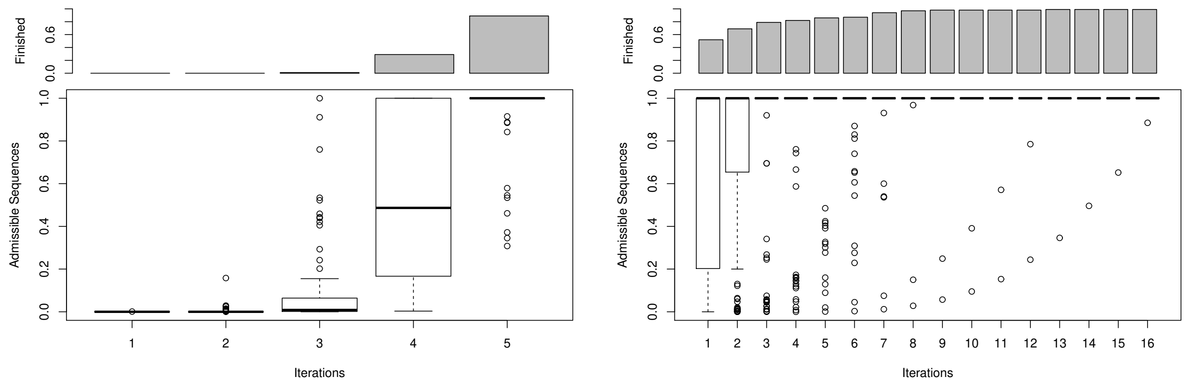

Figure 9: Relative numbers of finished instances and admissible sequences at each iteration of the seed generation procedure for the benchmark set `3str`. The left illustrates the procedure for targeting just the GC% in the precomputation step; this step is used to learn instance-specific transformations of approximate energies to the Turner model from the generated sample. The right plots illustrates generating samples targeting GC% and the Turner energies.

F Parameters for the base pair and the stacking energy model

We trained parameters for two RNA energy models to approximate the Turner energy model, as implemented in the `ViennaRNA` package. In the *base pair model*, the total energy of a sequence S for an RNA structure R is given as sum of base pair energies, where we consider six types of base pairs distinguishing by the bases A-U, C-G or G-U (symmetrically) and between stacked and non-stacked base pairs; here, we consider base pairs $(i, j) \in R$ *stacked* iff $(i+1, j-1) \in R$, otherwise *non-stacked*. In the *stacking model*, our features are defined by the stacks, i.e. pairs (i, j) and $(i+1, j-1)$ which both occur in R; we distinguish 18 types based on $S_i, S_j, S_{i+1}, S_{j-1}$ (i.e. all combinations that allow canonical base pairs; the configurations $S_i, S_j, S_{i+1}, S_{j-1}$ and $S_{i+1}, S_{j-1}, S_j, S_i$ are symmetric).

Both models describe the energy assigned to a pair of sequence and structure in linear dependency of the number of features and their weights. We can thus train weights for linear predictors of the Turner energy in both models.

For this purpose, we generated 5000 uniform random RNA sequences of random lengths between 100 and 200. For each sample, we predict the minimum free energy and corresponding structure using the ViennaRNA package; then, we count the distinguished features (i.e., base pair or stack types). The parameters are estimated fitting linear models without intercept (`R` function `lm`). For both models, `R` reports an adjusted R-squared value of 0.99. The resulting parameters are reported in Tables 1 and 2.

For validating the trained parameters, we generate a second independent test set of random RNA sequences in the same way. Fig. 10 shows correlation plots for the trained parameters in the base pair and stacking models for predicting the Turner energies in the test set with respective correlations of 0.95 an 0.94.

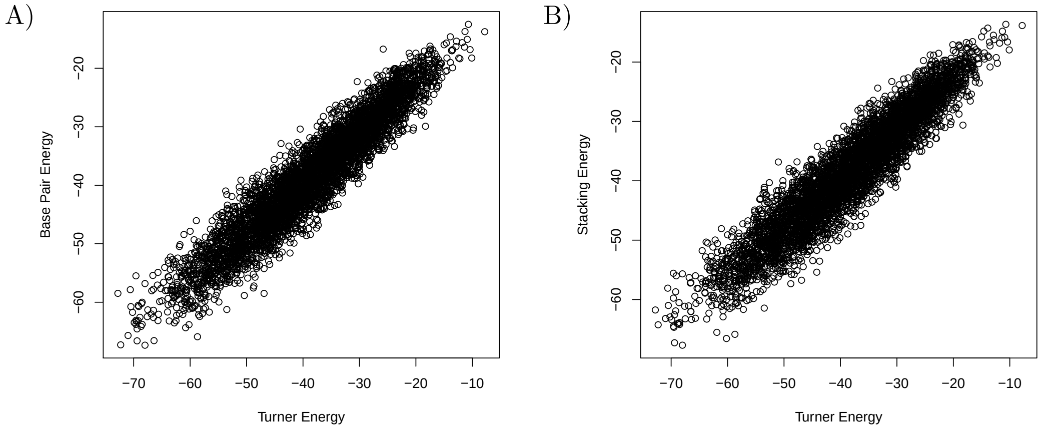

Figure 10: Validation of the trained parameters for the A) base pair model B) stacking model for predicting energies of the independently sampled sequences in the test set. We show correlation plots against the Turner energies reported by the ViennaRNA package.

G Tree decomposition with hyper-dependencies

Our implementation relies on external tools for the tree decomposition, which do not directly support tree decomposition of hyper-graphs. Therefore it is not immediately obvious how to obtain a suitable tree decomposition, such that even multi-ary contributions are respected and a cluster tree can be generated.

Crucially, one can show that, as long as certain vertices of a graph G from a clique, there exists at least one node in the tree decomposition of G that contains all such vertices. This allows us to generate suitable tree decompositions, which respect the hyper-edges of our dependency graph, by expanding the hyper-edges to cliques, and then calling the tree-decomposer on the resulting (non-hyper-)graph.

While the result is certainly part of folklore, we emphasize the importance for the generality of this work and present a brief direct proof for convenience.

Lemma 1 *Let $G = (V, E)$ be an undirected graph, and (T, χ) be a tree decomposition for G. For each clique $C \subseteq V$, there exists a node $u \in T$ such that $C \subseteq \chi(u)$.*

Table 1: Trained weights for the base pair energy model.

non-stacked			stacked		
AU	CG	GU	AU	GC	GU
1.26630	-0.09070	0.78566	-0.52309	-2.10208	-0.88474

Proof. Root T at an arbitrary tree node $r \in T$, which induces a parent/child relation between tree nodes. We say a tree node u *represents* the node $v \in V$ (respectively, the set of nodes $X \subseteq V$) iff $v \in \chi(u)$ ($X \subseteq \chi(u)$). Moreover, denote by $\text{tverts}(u) \subseteq V$ the vertices represented by the nodes of the subtree of u, i.e.

$$\text{tverts}(u) := \chi(u) \cup \bigcup_{\text{children } u' \text{ of u}} \text{tverts}(u').$$

Assume there is no node in T that represents C.

Let u denote the node in T with maximal distance to the root r, such that still $C \subseteq \text{tverts}(u)$. Since there is no single node u that represents C, this maximal node is unique, since if a node u, $C \subseteq \text{tverts}(u)$, has two subtrees that cover C, then u must represent C due to property 2 of Def. 1.

Let C' be $C - \chi(u)$. C' cannot be empty, since otherwise u represents Ci. For exactly one of the children u' of u, $C' \subseteq \text{tverts}(u')$, since otherwise for some node $v' \in C'$, the subtree of the nodes representing v' would be disconnected (property 2 of Def. 1). Now, pick some $v' \in C'$ and some $v'' \in C - C'$, which must be represented by u or some child u'' of u. In both cases, there cannot be any node in T that represents the edge $\{v', v''\} \subseteq C$, since v' can only be represented by nodes in the subtree of u'. Contradiction. □

This result implies that classic tree decomposition algorithms and, more importantly, implementations can be directly re-used to produce decompositions that capture energy models of arbitrary complexity, functions of arbitrary arity. Indeed, it suffices to add a clique, involving the parameters of the function, and Lemma 1 guarantees that the tree decomposition will feature one node to which the function can be associated.

H Correctness of the FPT partition function algorithm

Theorem 2 (Correctness of Alg. 1) *As computed by Alg. 1 for cluster tree* (T, χ, ϕ), *the messages* $m_{u \to v}$, *for all edges* $u \to v \in T$, *yield the partition functions of subtree of* u *for the partial sequences* $\bar{S} \in \mathcal{PS}(\text{sep}(u, v))$, *i.e. the messages satisfy*

$$m_{u \to v}(\bar{S}) = \sum_{\bar{S}' \in \mathcal{PS}(\chi(T_r(u)) - \text{sep}(u,v))} \prod_{f \in \phi(T_r(u))} \exp(-f(\bar{S}' \cup \bar{S})), \tag{1}$$

where $\chi(T_r(u))$ *denotes all* χ*-assigned positions of nodes in* $T_r(u)$*; respectively* $\phi(T_r(u))$*; all* ϕ*-assigned functions.*

Proof. Note that in more concise notation, Alg. 1 computes messages such that

$$m_{u \to v}(\bar{S}) := \sum_{\bar{S}' \in \mathcal{PS}(\text{diff}(u \to v))} \prod_{f \in \phi(u)} \exp(-f(\bar{S}' \cup \bar{S})) \prod_{(w \to u) \in T} m_{w \to u}(\bar{S}' \cup \bar{S}). \tag{2}$$

Proof by induction on T. If u is a leaf, $\chi(r) = \chi(T_r(u))$, there are no messages sent to u, and $\phi(u) = \phi(T_r(u))$; implying Eq. (1). Otherwise, since the algorithm traverses edges in postorder, u received from its children $w_1, \ldots, w_q$ the messages $m_{w_1 \to u}, \ldots, m_{w_q \to u}$, which satisfy Eq. (1) (induction hypothesis). Let

Table 2: Trained weights for the stacking energy model (the rows specify S_i, S_j; the columns, S_{i+1}, S_{j-1}; we do not show the symmetric weights).

	AU	CG	GC	GU	UA	UG
AU	-0.18826	-1.13291	-1.09787	-0.38606	-0.26510	-0.62086
CG	-1.11752	-2.23740	-1.89434	-1.22942	-1.10548	-1.44085
GU	-0.55066	-1.26209	-1.58478	-0.72185	-0.49625	-0.68876

$\bar{S} \in \mathcal{PS}(\text{sep}(u,v))$; then, $m_{u\to v}(\bar{S})$ is computed by the algorithm according to Eq. (2). We rewrite as follows

$$\sum_{\bar{S}'\in\mathcal{PS}(\text{diff}(u\to v))} \prod_{f\in\phi(u)} \exp(-f(\bar{S}'\cup\bar{S})) \prod_{(w\to u)\in T} m_{w\to u}(\bar{S}'\cup\bar{S})$$

$$= \sum_{\bar{S}'\in\mathcal{PS}(\chi(u)-\text{sep}(u,v))} \prod_{f\in\phi(u)} \exp(-f(\bar{S}'\cup\bar{S})) \prod_{i=1}^{q} m_{w_i\to u}(\bar{S}'\cup\bar{S})$$

$$=_{IH} \sum_{\bar{S}'\in\mathcal{PS}(\chi(u)-\text{sep}(u,v))} \prod_{f\in\phi(u)} \exp(-f(\bar{S}'\cup\bar{S})) \prod_{i=1}^{q} \sum_{\bar{S}''\in\mathcal{PS}(\chi(T_r(w_i))-\text{sep}(w_i,u))} \prod_{f\in\phi(T_r(w_i))} \exp(-f(\bar{S}''\cup\bar{S}'\cup\bar{S}))$$

$$=_{*} \sum_{\bar{S}'\in\mathcal{PS}(\chi(u)-\text{sep}(u,v))} \sum_{\bar{S}''\in\mathcal{PS}(\bigcup_{i=1}^{q}\chi(T_r(w_i))-\text{sep}(w_i,u))} \prod_{f\in\phi(u)} \exp(-f(\bar{S}'\cup\bar{S})) \prod_{f\in\phi(T_r(w_i))} \exp(-f(\bar{S}''\cup\bar{S}'\cup\bar{S}))$$

$$=_{(**)} \sum_{\bar{S}'\in\mathcal{PS}(\chi(T_r(u))-\text{sep}(u,v))} \prod_{f\in\phi(T_r(u))} \exp(-f(\bar{S}'\cup\bar{S}))$$

To see (*) and (**), we observe:

- The sets $\chi(u)-\text{sep}(u,v)$ and $\chi(T_r(w_i))-\text{sep}(w_i,u)$ are all disjoint due to Def. 1, property 2. First, this property implies that any shared position between the subtrees of w_i and w_j must be in $\chi(w_i)$, $\chi(w_j)$ and $\chi(u)$, thus the positions of $\chi(T_r(w_i)) - \text{sep}(w_i,u)$ are disjoint. Second, if a position $\chi(T_r(w_i))$ occurs in $\chi(u)$, it must occur in $\chi(w_i)$ and consequently in $\text{sep}(u,v)$.
- The union of the sets $\chi(u) - \text{sep}(u,v)$ and $\chi(T_r(w_i)) - \text{sep}(w_i,u)$ is $\chi(T_r(u)) - \text{sep}(u,v))$.

□

I General complexity of the partition function computation by cluster tree elimination and generation of samples

Proposition 3 *Given a cluster tree (T,χ,ϕ), $T=(V,E)$ of the set of contributions $\mathcal{F}$ with treewidth w and maximum separator size s, computing the partition function by Algorithm 1 takes $O((|F|+|V|)\cdot 4^{w+1})$ time and $O(|V|4^s)$ space (for storing all messages). The sampling step has time complexity of $O((|F|+|V|)\cdot 4^D)$ per sample.*

Proof (Proposition 3). Let d_u denote the degree of node u in T, s_u is the size of the separator between u and its parent in T rooted at r. For each node in the cluster tree, Alg. 1 computes one message by combining $(|\phi(u)|+d_u-1)$ functions, each time enumerating $4^{|\chi(u)|}$ combinations; Alg. 2 computes $4^{|\chi_u|-s_u}$ partition functions each time combining $(|\phi(u)|+d_u-1)$ functions. $4^{|\chi(u)|}$ is bound by 4^{w+1} and $4^{|\chi_u|-s_u}$ by 4^D; moreover $\sum_{u\in V}(|\phi(u)|+d_u-1) = |F|+|V|-1$. □

J Exploiting constraint consistency to reduce the complexity

While Alg. 1 computes messages values for *all* possible combinations of nucleotides for the positions in a node, we observe here that many such combinations are *not* required for computing all relevant partition functions. In particular, the algorithm can be restricted to consider only *valid* combinations, satisfying the (hard) constraints induced by valid base pairs.

The given structures in the RNA design problem impose constraints on the sequences for ensuring canonical (aka complementary) base pairing. These constraints can be exploited in a particularly simple and effective way to reduce the complexity. As previously noted by [2], the base pair complementarity induces a bi-partition of each connected component within the base pair dependency graph, such that the nucleotides

assigned to the two set of nodes in the partition are restricted to values in $\{\mathsf{A},\mathsf{G}\}$ and $\{\mathsf{C},\mathsf{U}\}$ respectively. We call a partial sequence *cc-valid*, iff its determined positions are consistent with such a separation for all determined positions of the same connected component.

One can now modify Alg. 1, on a tree decomposition (T,χ), such that the values of messages $m_{u\to v}$ are computed only for cc-valid partial sequences $\bar{S}\in\mathcal{PS}(\mathrm{sep}(u,v))$. Moreover, the loop over $\bar{S}'\in\mathcal{PS}(\mathrm{diff}(u\to v))$ is restricted, such that $\bar{S}\cup\bar{S}'$ are cc-valid. Analogous restrictions are then implemented in the sampling algorithm Alg. 2.

The correctness of the modified algorithm follows from the same induction argument, where the message computation over one node evaluates messages from its children only at cc-valid partial sequences. The result of this computation is a message, which corresponds to the partition function, restricted to cc-valid partial sequences. Since invalid sequences have infinite energy, they do not contribute to the partition function, and the partition function restricted to cc-valid sequences coincides with the initial one.

This restriction drastically improves the time complexity. Indeed, for any given node v, the original algorithm sends message for $\chi(v):=\bar{S}\cup\bar{S}'$ such that $\bar{S}\in\mathcal{PS}(\mathrm{sep}(u,v))$ and $\bar{S}'\in\mathcal{PS}(\mathrm{diff}(u\to v))$, while the modified algorithm only considers cc-valid assignments for $\chi(v)$. Remark that, in any connected component cc, assigning some nucleotide to a position reduces cc-valid assignments to (at most) two alternatives for each of the $|cc|-1$ remaining positions. It follows that, for a node v featuring positions from $\#cc(v)$ distinct connected components $\{cc_1,cc_2\ldots\}$ in the base pair dependency graph, the number of cc-valid assignments to positions in $\chi(v)$ is exactly

$$4^{\#cc(v)}\prod_{i=1}^{\#cc(v)}2^{|cc_i|-1}=2^{\#cc(v)}\,2^{|\chi(v)|}\in\Theta(2^{\#cc}\,2^{w+1}),$$

for a single node, where $\#cc$ is the total number of connected components in the base pair dependency graph, and w is the tree-width of the tree decomposition T. Since the number of nodes in T is in $\Theta(n)$, and the number of atomic energy contributions associated with k structures is in $\mathcal{O}(k\,n)$, then the overall complexity grows like $\Theta(n\,k\,2^{\#cc}\,2^{w+1})$.

Finally, we remark that even stronger time savings could be possible in practice, since cc-valid partial sequences can still violate complementarity constraints, e.g. by assigning C and A to positions in different sets of a partition, thus satisfying the bi-partition constraints, where the positions base pair directly, rendering the partial sequence invalid. Moreover, applications of the sampling framework can introduce additional constraints that further reduce the number of valid partial subsequences. However, exploiting all such constraints, in a complete and general way, would likely cause significant implementation overhead, while not significantly improving the asymptotic complexity.

K Monotonicity of the partial derivatives within weight calibration

In weighted distributions, one witnesses a fairly predictable impact of the variation of any weight π_ℓ over the expected value $\mathbb{E}(E_\ell\mid\boldsymbol{\pi})$, $\boldsymbol{\pi}:=(\pi_1\cdots\pi_k)$, of the free energy E_ℓ of structure ℓ. Let us first remind the probability of a sequence S in the weighted distribution

$$\mathcal{B}_{\boldsymbol{\pi}}(S)=\prod_{i=1}^{k}\pi_i^{-E_i(S)},\qquad \mathcal{Z}_{\boldsymbol{\pi}}=\sum_{S'}\mathcal{B}_{\boldsymbol{\pi}}(S')\qquad\text{and}\qquad \mathbb{P}(S\mid\boldsymbol{\pi})=\frac{\mathcal{B}_{\boldsymbol{\pi}}(S)}{\mathcal{Z}_{\boldsymbol{\pi}}}.$$

Remind also the definition of the expectation of $E_\ell(S)$, for S a Boltzmann-distributed sequence:

$$\mathbb{E}(E_\ell\mid\boldsymbol{\pi})=\sum_{S}E_\ell(S)\cdot\mathbb{P}(S\mid\boldsymbol{\pi}).$$

We first remark that the partial derivative of $\mathcal{B}$ yields

$$\frac{\partial \mathcal{B}_{\boldsymbol{\pi}}(S)}{\partial \pi_\ell} = -E_\ell(S) \cdot \pi_\ell^{-E_\ell(S)-1} \prod_{\substack{i=1\\ i\neq \ell}}^{k} \pi_i^{-E_i(S)} = \frac{-E_\ell(S)}{\pi_\ell} \cdot \mathcal{B}_{\boldsymbol{\pi}}(S) = -E_\ell(S) \cdot \mathbb{P}(S \mid \boldsymbol{\pi}) \cdot \frac{\mathcal{Z}_{\boldsymbol{\pi}}}{\pi_\ell}$$

while the partial derivative of $\mathcal{Z}$ gives

$$\frac{\partial \mathcal{Z}_{\boldsymbol{\pi}}(S)}{\partial \pi_\ell} = \sum_{S'} -E_\ell(S') \cdot \mathbb{P}(S' \mid \boldsymbol{\pi}) \cdot \frac{\mathcal{Z}_{\boldsymbol{\pi}}}{\pi_\ell} = -\mathbb{E}(E_\ell \mid \boldsymbol{\pi}) \cdot \frac{\mathcal{Z}_{\boldsymbol{\pi}}}{\pi_\ell}.$$

From these expressions, we conclude that

$$\begin{aligned}
\frac{\partial \mathbb{E}(E_\ell \mid \boldsymbol{\pi})}{\partial \pi_\ell} &= \sum_S E_\ell(S) \cdot \frac{\partial \mathbb{P}(S \mid \boldsymbol{\pi})}{\partial \pi_\ell} \\
&= \sum_S E_\ell(S) \left(\frac{\frac{\partial \mathcal{B}_{\boldsymbol{\pi}}(S)}{\partial \pi_\ell}}{\mathcal{Z}_{\boldsymbol{\pi}}} - \frac{\frac{\partial \mathcal{Z}_{\boldsymbol{\pi}}}{\partial \pi_\ell} \cdot \mathcal{B}_{\boldsymbol{\pi}}(S)}{\mathcal{Z}_{\boldsymbol{\pi}}^2} \right) \\
&= \sum_S E_\ell(S) \left(\frac{-E_\ell(S) \cdot \mathbb{P}(S \mid \boldsymbol{\pi}) \cdot \frac{\mathcal{Z}_{\boldsymbol{\pi}}}{\pi_\ell}}{\mathcal{Z}_{\boldsymbol{\pi}}} + \frac{\mathbb{E}(E_\ell \mid \boldsymbol{\pi}) \cdot \frac{\mathcal{Z}_{\boldsymbol{\pi}}}{\pi_\ell} \cdot \mathcal{B}_{\boldsymbol{\pi}}(S)}{\mathcal{Z}_{\boldsymbol{\pi}}^2} \right) \\
&= \sum_S -\frac{E_\ell(S)^2 \cdot \mathbb{P}(S \mid \boldsymbol{\pi})}{\pi_\ell} + \frac{\mathbb{E}(E_\ell \mid \boldsymbol{\pi})}{\pi_\ell} \sum_S E_\ell(S) \cdot \mathbb{P}(S \mid \boldsymbol{\pi}) \\
&= -\frac{\mathbb{E}(E_\ell^2 \mid \boldsymbol{\pi}) - \mathbb{E}(E_\ell \mid \boldsymbol{\pi})^2}{\pi_\ell}
\end{aligned}$$

In the numerator of the above expression, one recognizes the variance of the distribution of E_ℓ. Remark that a variance is always non-negative and, in our case, also strictly positive for $\boldsymbol{\pi}$, $\pi_\ell > 0$, as soon as there exist at least two distinct sequences S and S' such that $E_\ell(S) \neq E_\ell(S')$. Moreover, weights are always positive so the partial derivative in π_ℓ is always positive. Ultimately, it is always possible to increase (resp. decrease) the expected free energy of a structure by simply decreasing (resp. increasing) its weight, supporting our weight optimization procedure.

L Approximate counting and random generation

In fact, not only is #BIS a reference problem in counting complexity, but it is also a landmark problem with respect to the complexity of approximate counting problems. In this context, it is the representative for a class of #BIS-hard problems [3] that are easier to approximate than #SAT, yet are widely believed not to admit any Fully Polynomial-time Randomized Approximation Scheme. Recent results reveal a surprising dichotomy in the behavior of #BIS: it admits a Fully Polynomial-Time Approximation Scheme (FPTAS) for graphs of max degree ≤ 5 [4], but is as hard to approximate as the general #BIS problem on graphs of degree ≥ 6 [5]. In other words, there is a clear threshold, in term of the max degree, separating (relatively) easy instances from really hard ones.

Additionally, let us note that, from the classic Vizing Theorem, any bipartite graph G having maximum degree Δ can be decomposed in polynomial time in exactly Δ matchings. Any such matching can be reinterpreted as a secondary structure, possibly with crossing interactions (*pseudoknots*). These results have two immediate consequences for the pseudoknotted version of the multiple design counting problem.

Corollary 4 (as follows from [4]) *The number of designs compatible with $m \leq 5$ pseudoknotted RNA structures can be approximated within any fixed ratio by a deterministic polynomial-time algorithm.*

Corollary 5 (as follows from [5]) *As soon as the number of pseudoknotted RNA structures strictly exceeds 5, #Designs is as hard to approximate as #BIS.*

It is worth noting that the #P hardness of #Designs does not immediately imply the hardness of generating a valid design uniformly at random, as demonstrated constructively by Jerrum, Valiant and Vazirani [6]. However, in the same work, the authors establish a strong connection between the complexity of approximate counting and the uniform random generation. Namely, they showed that, for problems associated with self-reducible relations, approximate counting is equally hard as (almost) uniform random generation. We conjecture that the (almost) uniform sampling of sequences from multiple structures with pseudoknots is in fact #BIS-hard as soon as the number of input structures strictly exceeds 5, as indicated by [7], motivating even further our parameterized approach.

M Contribution of terms to the MultiDefect score

Figure 11 breaks down the multi-defect score of Eq. (3), which is used to evaluate the quality of multi-target designs. Recall that the total score is composed of the term $\frac{1}{k}\sum_{\ell=1}^{k}(E(S,R_\ell) - \mathrm{G}(S))$, which evaluates the distances of the energies of target structures to the minimum free energy (Distance to MFE), and the term $\frac{1}{2\binom{k}{2}}\sum_{1\leq \ell<j\leq k}|E(S,R_\ell)-E(S,R_j)|$, which evaluates the similarity of the target structure energies of the target structures (Heterogeneity).

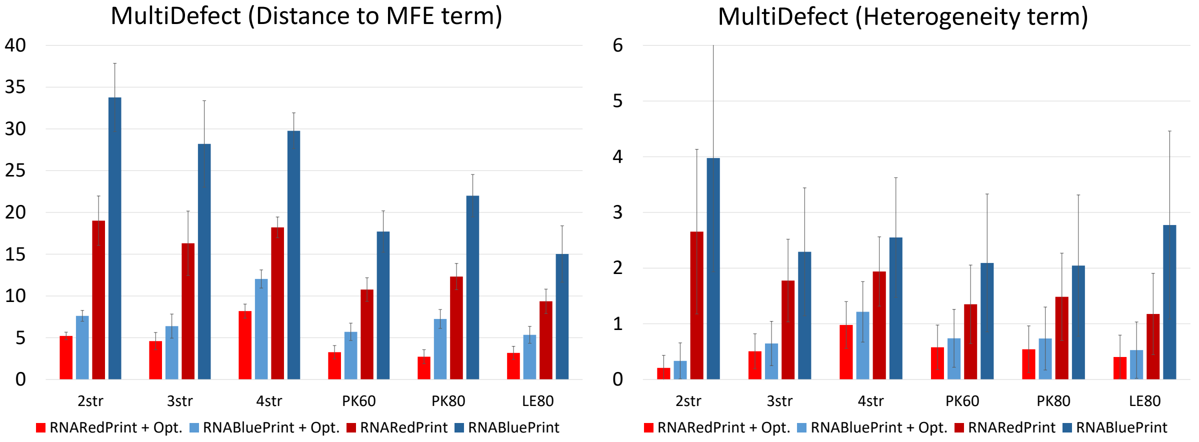

Figure 11: Break-down of average MultiDefect values into their two components.

N Detailed results of the negative design benchmark

N/A values correspond to data that could not be obtained by the time of this submission for two main reasons: In the case of the initial sampling (left columns), they correspond to instances which, in conjunction with an expressive energy model, resulted in very high tree-width, leading to unreasonable memory requirements incompatible with our available computing resources; The case of missing values after optimization (right columns), they indicate situations where the initial optimization took too long ($\approx$ 1 day) and was killed.

N.1 RNAtabupath (2 structures)

	Boltzmann		Uniform			Boltzmann Optimized		Uniform Optimized		
Name	Mean	StdDev	Mean	StdDev	Δ	Mean	StdDev	Mean	StdDev	Δ_{opt}
alpha_operon	17.15	4.06	25.86	5.97	8.70	2.13	0.83	2.36	0.88	0.23
amv	14.34	2.87	27.26	5.65	12.92	3.75	0.90	4.27	1.08	0.52

attenuator	10.80	3.03	21.44	4.77	10.64	1.37	0.53	1.81	0.80	0.44
dsrA	11.81	2.58	18.23	4.62	6.42	3.75	0.94	3.80	0.95	0.06
hdv	22.00	4.81	35.48	6.68	13.47	3.64	1.14	4.57	1.48	0.92
hiv	41.07	7.02	73.00	9.40	31.92	17.07	3.26	30.06	5.34	12.99
ms2	13.36	3.72	19.79	4.55	6.43	2.36	0.79	2.66	0.97	0.30
rb1	18.40	4.48	37.38	7.14	18.98	2.63	0.92	3.82	1.31	1.19
rb2	16.59	4.10	25.17	5.85	8.58	2.81	0.84	3.07	0.97	0.26
rb3	22.43	4.57	41.18	7.09	18.75	2.77	0.82	5.05	1.52	2.27
rb4	26.11	4.32	47.89	6.80	21.77	7.00	1.15	11.80	1.82	4.80
rb5	30.77	5.47	54.24	7.76	23.48	6.01	1.96	9.22	2.78	3.21
ribD	49.88	7.09	81.27	9.07	31.39	20.35	3.27	30.75	4.33	10.39
s15	10.93	2.94	18.37	4.53	7.44	1.95	0.67	2.12	0.77	0.16
sbox	24.93	4.63	50.03	8.04	25.10	6.28	1.56	9.08	2.28	2.80
spliced	9.65	2.96	17.60	4.39	7.95	2.15	0.46	2.41	0.59	0.26
sv11	20.95	4.63	36.83	6.91	15.88	3.04	0.88	4.20	1.12	1.17
thim	29.23	5.43	48.32	6.95	19.09	8.58	1.86	12.12	2.54	3.54
Mean	21.69	4.37	37.74	6.45	16.05	5.42	1.27	7.95	1.75	2.53

N.2 RNADesign (3 structures)

Name	Boltzmann Mean	Boltzmann StdDev	Uniform Mean	Uniform StdDev	Δ	Boltzmann Optimized Mean	Boltzmann Optimized StdDev	Uniform Optimized Mean	Uniform Optimized StdDev	Δ_{opt}
sq100	20.73	5.02	31.78	5.46	11.05	5.81	1.53	8.33	1.92	2.53
sq10	19.73	5.27	26.62	5.59	6.89	3.50	0.81	3.88	0.94	0.38
sq11	19.46	4.73	27.76	5.69	8.30	2.81	0.77	3.31	0.89	0.49
sq12	17.09	3.57	22.62	4.98	5.53	2.70	0.65	2.79	0.68	0.09
sq13	18.55	3.96	29.95	5.56	11.40	10.24	2.18	16.63	3.13	6.39
sq14	21.52	4.25	37.82	5.55	16.31	7.25	1.63	9.90	2.10	2.65
sq15	21.93	3.77	36.32	5.13	14.39	11.15	1.75	15.49	2.34	4.33
sq16	15.86	4.23	27.70	5.58	11.84	2.51	0.69	2.96	0.94	0.45
sq17	20.66	3.69	33.62	5.33	12.97	5.83	1.08	7.36	1.49	1.52
sq18	19.70	3.86	35.26	5.13	15.57	5.83	1.41	7.97	2.00	2.14
sq19	14.78	3.37	28.64	5.34	13.86	3.84	0.77	4.66	1.04	0.82
sq1	14.75	3.75	21.89	5.48	7.14	2.02	0.65	2.20	0.69	0.18
sq20	17.41	3.81	27.90	4.93	10.48	3.93	1.04	5.18	1.48	1.24
sq21	21.66	4.03	36.27	5.16	14.60	8.28	1.82	11.41	2.40	3.12
sq22	17.69	4.13	31.57	5.45	13.89	5.66	1.51	10.02	2.50	4.36
sq23	19.35	3.61	32.38	5.40	13.03	12.05	1.85	18.69	3.18	6.65
sq24	17.96	4.03	27.92	5.30	9.96	3.59	0.93	4.25	1.10	0.66
sq25	17.65	5.10	26.65	6.11	9.00	1.35	0.34	1.45	0.40	0.10
sq26	17.64	4.51	24.46	5.64	6.82	3.11	0.62	3.38	0.68	0.27
sq27	18.08	3.57	28.51	5.30	10.43	7.55	1.49	10.96	2.38	3.41
sq28	14.90	3.66	29.35	5.99	14.46	2.32	0.49	2.66	0.62	0.34
sq29	17.65	3.69	26.95	5.08	9.30	4.44	0.98	5.22	1.21	0.78
sq2	22.38	4.17	37.25	5.15	14.87	10.34	1.71	14.32	2.35	3.99
sq30	19.14	4.08	31.75	5.54	12.62	6.06	1.61	8.99	2.19	2.93
sq31	19.94	3.83	31.92	5.20	11.98	5.12	1.19	6.34	1.55	1.22
sq32	22.06	3.99	31.65	5.23	9.59	5.24	1.29	6.84	1.76	1.60
sq33	15.21	4.28	22.75	5.52	7.54	1.78	0.43	2.08	0.63	0.30
sq34	25.01	4.14	37.46	5.03	12.46	12.46	1.84	18.73	2.57	6.27
sq35	19.12	4.11	32.72	5.35	13.61	5.90	1.25	7.96	1.82	2.06

sq36	12.72	2.81	26.76	5.39	14.04	3.19	0.77	3.61	0.94	0.41
sq37	18.72	3.83	32.44	5.62	13.72	3.24	0.90	4.21	1.25	0.98
sq38	14.73	3.61	29.02	5.29	14.29	2.79	0.86	3.70	1.22	0.91
sq39	22.75	3.96	33.06	4.98	10.30	9.20	1.76	11.87	2.33	2.67
sq3	20.34	4.02	36.86	5.60	16.52	4.69	1.19	6.30	1.72	1.61
sq40	19.52	4.33	31.76	5.41	12.24	4.05	0.94	4.96	1.19	0.91
sq41	14.44	3.48	27.85	5.57	13.41	3.33	0.71	3.91	0.88	0.58
sq42	18.68	3.89	35.20	5.55	16.51	5.32	1.24	6.96	1.56	1.64
sq43	17.32	3.94	31.63	5.38	14.31	3.53	0.89	4.27	1.15	0.74
sq44	19.38	3.66	31.75	5.09	12.37	5.52	1.29	7.26	1.60	1.74
sq45	18.49	4.74	27.40	5.53	8.90	2.54	0.67	2.84	0.74	0.30
sq46	17.97	3.84	28.83	5.09	10.86	4.45	0.90	5.41	1.20	0.96
sq47	15.22	3.86	28.23	5.82	13.01	3.02	0.78	3.45	0.85	0.43
sq48	18.89	4.14	36.27	5.78	17.38	4.69	1.08	6.71	1.67	2.02
sq49	20.19	4.73	27.24	5.39	7.05	3.15	0.76	3.66	0.95	0.51
sq4	19.32	4.73	29.25	5.34	9.93	2.36	0.79	3.24	1.15	0.88
sq50	16.92	3.83	28.43	5.35	11.51	3.15	0.73	3.54	0.86	0.38
sq51	22.17	4.07	36.07	5.20	13.90	11.56	1.79	16.57	2.88	5.01
sq52	19.33	3.94	31.04	5.20	11.71	11.04	2.21	16.25	3.04	5.21
sq53	13.65	3.69	24.48	5.46	10.83	1.65	0.39	1.88	0.53	0.23
sq54	18.25	3.67	33.99	5.57	15.74	8.15	1.57	12.35	2.59	4.19
sq55	22.12	4.75	30.11	5.21	8.00	4.59	0.82	5.43	1.13	0.83
sq56	17.85	3.90	31.69	5.46	13.84	4.22	0.75	4.84	0.93	0.62
sq57	16.39	4.07	28.48	5.45	12.08	1.96	0.58	2.28	0.71	0.32
sq58	12.73	2.86	32.97	5.80	20.24	3.77	0.82	4.89	1.22	1.11
sq59	20.45	4.54	33.02	5.57	12.57	6.05	1.50	8.34	2.08	2.29
sq5	9.90	2.70	17.94	4.93	8.04	1.29	0.33	1.41	0.39	0.12
sq60	22.73	4.85	36.86	5.46	14.13	6.90	1.62	11.07	2.33	4.17
sq61	14.34	3.75	30.82	6.03	16.48	3.07	0.76	3.83	1.01	0.76
sq62	21.12	5.04	35.27	5.35	14.15	9.76	1.67	16.93	2.79	7.17
sq63	13.38	3.56	27.20	5.63	13.82	1.58	0.36	1.85	0.53	0.27
sq64	19.19	3.90	34.88	5.24	15.69	6.10	1.53	9.45	2.29	3.35
sq65	19.39	4.58	27.51	5.15	8.12	13.95	2.94	19.40	3.61	5.45
sq66	18.14	3.71	30.79	5.12	12.65	6.93	1.42	10.05	2.03	3.12
sq67	20.62	3.40	37.06	5.18	16.44	16.11	1.97	27.21	3.40	11.10
sq68	23.09	4.33	33.33	5.08	10.24	6.99	1.55	9.23	1.94	2.24
sq69	18.76	3.63	35.06	5.21	16.29	10.77	1.94	18.38	3.00	7.61
sq6	20.48	3.75	38.48	5.48	18.00	5.55	1.32	8.63	2.04	3.08
sq70	13.52	3.25	31.06	5.52	17.54	2.59	0.80	3.80	1.17	1.21
sq71	20.04	4.04	32.71	5.39	12.67	5.57	1.06	6.61	1.30	1.04
sq72	14.96	3.15	24.48	4.94	9.51	2.02	0.57	2.49	0.84	0.47
sq73	14.97	3.75	28.44	5.56	13.47	2.34	0.69	3.10	1.04	0.75
sq74	17.77	4.06	30.13	5.30	12.36	4.12	0.79	5.03	1.04	0.91
sq75	19.63	4.00	31.19	5.19	11.56	5.63	1.26	7.52	1.70	1.89
sq76	19.91	3.93	32.44	5.12	12.53	9.12	1.92	13.89	2.39	4.78
sq77	18.70	3.45	34.83	5.21	16.12	6.01	1.25	8.61	1.75	2.61
sq78	18.29	3.50	32.94	6.01	14.66	5.39	1.12	7.07	1.51	1.68
sq79	21.66	4.74	30.73	5.47	9.07	13.10	2.89	17.86	3.45	4.75
sq7	20.44	3.92	32.95	5.41	12.52	5.51	1.70	6.68	1.90	1.17
sq80	15.57	3.93	28.29	5.30	12.71	1.85	0.53	2.41	0.88	0.56
sq81	21.44	4.58	29.92	5.44	8.48	3.74	0.95	4.67	1.33	0.93
sq82	8.94	2.38	19.89	5.36	10.94	1.39	0.21	1.47	0.28	0.09
sq83	14.12	3.47	28.92	5.45	14.80	2.77	0.76	3.52	1.01	0.75
sq84	15.63	3.94	24.12	5.26	8.49	2.54	0.47	2.85	0.65	0.31

sq85	15.54	3.43	26.09	5.18	10.55	2.19	0.62	2.70	0.82	0.51
sq86	21.77	5.09	34.21	5.77	12.43	4.66	1.21	5.92	1.51	1.26
sq87	16.55	3.87	32.65	5.81	16.10	3.50	0.62	4.14	0.86	0.64
sq88	20.10	5.08	33.45	5.86	13.34	2.50	0.72	3.17	0.99	0.68
sq89	14.74	4.28	26.15	5.59	11.41	1.79	0.50	2.01	0.62	0.23
sq8	15.80	4.14	30.34	6.02	14.55	2.85	0.79	3.85	1.15	1.00
sq90	15.30	3.88	29.83	5.80	14.53	2.10	0.64	2.90	1.06	0.79
sq91	17.38	3.99	27.19	5.08	9.81	3.69	1.11	4.48	1.31	0.79
sq92	19.15	3.54	33.07	5.34	13.91	4.77	1.11	6.83	1.78	2.06
sq93	19.54	4.22	29.17	5.65	9.63	3.29	0.80	3.86	1.03	0.57
sq94	14.79	3.26	27.05	5.43	12.26	3.98	0.77	4.66	0.98	0.68
sq95	17.58	3.67	33.42	5.38	15.84	12.86	2.20	22.76	3.84	9.90
sq96	15.44	3.87	31.62	5.68	16.18	2.69	0.67	3.52	1.02	0.83
sq97	18.38	3.98	29.22	5.03	10.83	4.31	1.13	5.32	1.42	1.01
sq98	18.83	4.59	29.30	5.42	10.47	2.78	0.73	3.59	0.97	0.80
sq99	19.61	4.14	30.95	5.28	11.34	3.94	0.98	4.89	1.33	0.95
sq9	17.05	4.26	26.15	5.72	9.11	1.66	0.44	1.79	0.55	0.13
Mean	18.10	3.98	30.49	5.41	12.39	5.10	1.10	7.04	1.52	1.94

N.3 RNADesign (4 structures)

	Boltzmann		Uniform			Boltzmann Optimized		Uniform Optimized		
Name	Mean	StdDev	Mean	StdDev	Δ	Mean	StdDev	Mean	StdDev	Δ_{opt}
sq100	21.70	4.41	33.19	4.93	11.49	12.32	2.52	17.14	3.10	4.82
sq10	20.66	5.16	27.00	5.24	6.34	6.63	1.91	8.02	2.05	1.39
sq11	22.23	4.23	30.73	5.31	8.50	6.18	1.30	7.70	1.70	1.52
sq12	18.30	3.55	23.38	4.93	5.08	3.78	0.57	3.94	0.64	0.17
sq13	21.01	3.57	31.66	4.93	10.65	15.61	1.75	21.61	2.69	6.00
sq14	22.46	3.09	39.77	5.28	17.31	17.59	2.28	25.04	3.21	7.45
sq15	22.66	3.72	36.31	5.01	13.65	17.42	2.41	26.80	3.34	9.38
sq16	18.22	4.12	28.54	5.33	10.32	3.29	0.76	3.78	0.90	0.50
sq17	23.04	4.01	35.87	5.28	12.82	12.63	2.37	18.43	2.86	5.79
sq18	21.20	3.49	35.78	5.39	14.57	8.24	1.53	10.75	2.02	2.51
sq19	15.36	2.73	32.12	4.95	16.76	13.30	1.67	23.64	3.41	10.34
sq1	15.14	3.83	22.32	5.28	7.18	2.53	0.56	2.66	0.56	0.13
sq20	16.75	3.09	29.25	4.74	12.50	9.37	1.60	13.40	2.41	4.03
sq21	23.98	3.72	38.18	5.14	14.20	16.47	2.08	23.31	3.16	6.83
sq22	20.38	3.81	32.90	5.12	12.53	8.58	1.42	12.39	2.03	3.81
sq23	18.39	3.36	32.82	5.34	14.43	12.09	1.81	19.58	3.26	7.50
sq24	20.05	4.10	29.72	5.29	9.67	5.45	1.10	7.28	1.68	1.84
sq25	21.48	5.35	28.83	5.78	7.35	2.85	0.63	3.30	0.77	0.45
sq26	20.83	4.63	27.39	5.25	6.56	4.17	0.76	4.80	0.96	0.63
sq27	17.14	3.48	29.41	5.24	12.27	7.56	1.42	11.67	2.34	4.12
sq28	23.50	4.48	33.84	6.07	10.35	5.61	1.10	6.69	1.39	1.07
sq29	18.14	3.65	27.22	4.85	9.08	5.47	0.88	6.26	1.17	0.78
sq2	22.16	3.44	36.10	4.90	13.94			24.62	2.30	24.62
sq30	19.40	3.76	32.40	4.95	13.00	8.77	1.58	12.39	2.19	3.62
sq31	24.97	3.80	32.68	5.19	7.72	8.71	1.28	13.05	2.15	4.34
sq32	19.62	3.76	31.03	4.97	11.41	5.47	1.23	7.57	1.71	2.10
sq33	19.51	4.25	27.65	5.12	8.14	4.30	0.96	5.37	1.16	1.07
sq34	25.15	3.97	37.58	4.90	12.43	14.29	1.86	20.62	2.68	6.33

sq35	21.87	3.93	35.50	5.73	13.63	9.63	1.52	13.80	2.31	4.17
sq36	11.65	2.59	26.29	5.48	14.64	3.30	0.63	3.86	0.90	0.56
sq37	25.80	4.48	36.19	4.90	10.39	15.78	2.11	23.46	2.85	7.68
sq38	17.74	3.43	32.11	5.14	14.37	4.95	0.98	6.20	1.38	1.25
sq39	21.54	3.83	32.74	5.21	11.20	9.77	1.72	13.10	2.29	3.33
sq3	20.19	4.06	39.87	5.23	19.68	14.54	2.18	26.21	3.32	11.67
sq40	18.68	4.18	33.41	5.62	14.73	4.29	0.95	5.49	1.30	1.20
sq41	15.86	3.46	30.06	5.30	14.19	5.52	0.95	6.59	1.17	1.08
sq42	21.79	3.25	38.72	5.11	16.93	14.45	2.03	23.27	3.45	8.82
sq43	23.21	3.99	33.89	5.33	10.68	7.16	1.30	9.08	1.76	1.92
sq44	21.65	3.59	33.47	4.99	11.83	8.02	1.47	10.91	1.97	2.89
sq45	19.01	4.79	27.79	5.58	8.78	3.48	0.66	3.81	0.83	0.33
sq46	20.08	3.60	30.69	4.89	10.61	7.76	1.30	9.95	1.73	2.18
sq47	17.29	3.73	29.94	5.26	12.65	4.47	1.00	5.23	1.20	0.76
sq48	28.78	4.03	38.24	5.57	9.46	16.85	1.71	25.59	3.19	8.73
sq49	19.19	4.75	27.33	5.42	8.14	3.14	0.76	3.65	0.86	0.52
sq4	19.71	4.46	29.44	5.30	9.73	3.37	0.89	4.26	1.18	0.89
sq50	19.36	4.11	30.60	5.49	11.24	4.98	0.81	5.73	0.99	0.75
sq51	24.16	2.87	38.68	5.02	14.52	20.73	1.51	28.05	2.50	7.32
sq52	21.40	3.37	33.80	5.43	12.40	16.23	2.28	25.12	3.70	8.90
sq53	15.81	3.50	28.75	5.32	12.94	4.20	0.99	6.20	1.53	2.00
sq54	18.16	3.45	35.10	4.86	16.93	15.41	2.16	26.23	3.39	10.82
sq55	22.47	4.43	31.94	5.15	9.47	10.91	2.50	15.20	2.91	4.28
sq56	21.11	4.12	35.25	5.93	14.15	12.20	2.06	18.65	2.91	6.44
sq57	18.94	3.91	30.86	5.51	11.92	3.69	0.74	4.50	0.90	0.81
sq58	19.68	2.97	35.80	5.41	16.12	9.76	1.44	16.74	2.69	6.98
sq59	20.35	3.75	33.08	5.20	12.73	17.54	3.02	24.34	3.91	6.81
sq5	11.60	2.54	19.61	5.12	8.01	1.90	0.38	2.13	0.49	0.23
sq60	21.97	4.37	38.39	5.44	16.42	17.46	2.56	29.58	3.88	12.12
sq61	19.07	3.46	34.14	5.24	15.07	7.04	1.32	10.28	1.97	3.24
sq62	25.71	5.09	34.50	4.88	8.79	12.06	1.87	17.32	2.60	5.26
sq63	17.21	3.45	30.71	5.37	13.50	5.47	1.33	7.39	1.83	1.92
sq64	19.89	3.52	35.51	4.96	15.62	13.87	2.03	22.12	3.10	8.24
sq65	18.69	4.06	29.18	5.34	10.50	12.71	2.30	17.66	2.97	4.96
sq66	17.50	3.40	30.59	5.16	13.09	8.67	1.59	13.61	2.42	4.94
sq67	22.63	4.21	36.73	5.06	14.10	17.08	2.27	26.85	3.33	9.77
sq68	24.93	4.36	35.00	5.13	10.07	12.42	1.87	17.96	2.39	5.54
sq69	22.48	3.16	36.07	4.91	13.59	19.69	1.64	24.53	2.08	4.84
sq6	22.32	3.77	40.27	5.41	17.95	6.16	1.36	9.46	2.08	3.29
sq70	16.97	4.07	31.84	5.51	14.87	3.60	0.84	5.09	1.32	1.49
sq71	19.34	4.07	31.81	5.24	12.47	5.14	0.95	6.26	1.20	1.12
sq72	16.60	3.01	29.22	4.92	12.62	6.11	1.17	7.67	1.57	1.55
sq73	18.64	3.44	30.86	5.16	12.23	12.22	2.59	21.15	4.05	8.93
sq74	17.24	3.88	31.19	5.47	13.95	12.62	3.37	21.49	4.11	8.87
sq75	18.44	3.60	31.25	5.19	12.82	7.25	1.35	11.19	2.21	3.94
sq76	20.76	3.38	33.74	4.94	12.98	16.76	2.27	25.18	3.38	8.42
sq77	21.92	3.29	35.83	5.26	13.91	10.39	1.64	13.96	2.25	3.58
sq78	23.60	3.17	36.23	5.47	12.64	19.10	2.58	26.07	3.61	6.96
sq79	24.77	4.43	32.49	5.23	7.71	17.83	2.85	23.78	3.64	5.95
sq7	19.94	3.54	32.78	5.29	12.84	5.35	1.64	6.59	1.89	1.24
sq80	18.63	3.88	29.75	5.27	11.12	2.84	0.64	3.64	1.01	0.80
sq81	23.57	3.63	36.02	5.24	12.45	17.61	2.27	25.76	3.17	8.15
sq82	13.21	3.25	25.45	5.00	12.24	3.01	0.68	4.06	1.00	1.05
sq83	14.37	3.21	31.96	5.41	17.59	3.85	0.89	5.55	1.31	1.70

sq84	19.05	3.89	29.35	5.00	10.31	5.20	0.99	6.38	1.32	1.18
sq85	17.32	3.91	30.80	5.43	13.48	10.64	2.01	17.53	2.93	6.89
sq86	25.19	4.04	36.79	5.66	11.60	8.98	1.94	14.14	2.62	5.16
sq87	16.91	3.89	32.84	5.34	15.94	4.86	0.75	6.08	1.18	1.22
sq88	22.92	4.99	35.87	5.65	12.95	5.21	0.97	6.58	1.30	1.37
sq89	17.52	3.91	29.24	5.45	11.72	4.12	0.87	4.83	1.11	0.71
sq8	25.56	4.49	33.42	5.21	7.86	9.60	1.55	12.55	2.05	2.94
sq90	18.67	3.62	33.21	5.33	14.54	5.41	1.01	7.26	1.62	1.85
sq91	18.00	3.83	26.82	4.85	8.82	8.10	1.80	11.08	2.41	2.98
sq92	24.32	3.52	37.62	4.84	13.30	20.06	2.17	29.08	3.47	9.02
sq93	22.63	4.66	33.40	5.25	10.77	11.48	2.12	16.37	3.00	4.88
sq94	14.71	3.25	28.06	5.39	13.35	3.76	0.76	4.51	0.97	0.75
sq95	23.77	4.11	36.93	5.11	13.17	16.41	2.12	23.98	2.67	7.57
sq96	17.20	3.70	33.01	5.46	15.81	5.90	1.14	8.09	1.71	2.19
sq97	19.78	3.85	30.01	5.09	10.23	6.14	1.24	7.45	1.60	1.31
sq98	21.39	4.33	31.13	5.16	9.73	4.74	0.78	5.67	1.03	0.94
sq99	20.33	4.39	29.97	5.07	9.64	4.03	1.07	4.95	1.28	0.92
sq9	18.86	4.22	29.51	5.22	10.65	3.94	0.90	5.06	1.35	1.11
Mean	20.15	3.83	32.32	5.23	12.17	9.17	1.51	13.25	2.13	4.17

N.4 Pseudoknots (PK60)

Name	Boltzmann Mean	Boltzmann StdDev	Uniform Mean	Uniform StdDev	Δ	Boltzmann Optimized Mean	Boltzmann Optimized StdDev	Uniform Optimized Mean	Uniform Optimized StdDev	Δ_{opt}
no10	8.77	3.14	17.74	4.46	8.96	1.13	1.19	4.39	1.88	3.26
no11	9.40	3.11	16.91	4.07	7.51	2.12	1.12	4.20	1.83	2.08
no12	10.81	3.09	21.95	4.54	11.14	3.61	1.05	6.74	2.01	3.13
no13	12.33	2.73	21.72	4.06	9.40	4.71	1.26	7.94	2.23	3.23
no14	9.51	3.00	19.13	4.27	9.62	2.23	0.98	5.23	1.76	3.00
no15	11.62	2.96	18.90	4.16	7.28	4.07	1.15	6.40	1.72	2.33
no16	13.14	3.09	19.67	4.19	6.53	4.03	1.17	6.36	1.77	2.33
no17	16.39	3.20	21.04	4.29	4.65	6.39	1.23	7.82	1.61	1.42
no18	11.41	3.37	18.95	4.41	7.55	1.80	1.75	4.86	2.11	3.06
no19	11.06	3.35	20.90	4.27	9.84	2.03	1.49	6.40	2.19	4.37
no1	11.88	2.85	22.33	4.39	10.45	3.78	0.99	7.58	1.93	3.80
no20	14.55	2.99	19.17	3.97	4.63	5.21	1.16	7.08	1.64	1.87
no21	15.73	3.62	22.73	4.36	7.01	6.25	1.36	8.29	1.83	2.04
no22	10.23	2.97	15.62	4.03	5.39	1.51	0.97	3.86	1.68	2.35
no23	11.64	2.11	16.35	4.40	4.71	3.85	1.04	4.86	1.35	1.01
no24	13.13	2.68	18.57	4.17	5.45	3.79	1.28	5.83	1.72	2.04
no25	5.91	2.46	16.02	4.18	10.11	0.26	0.72	2.95	1.59	2.69
no26	10.57	3.06	19.60	4.19	9.03	3.43	1.04	6.45	1.84	3.02
no27	14.49	2.46	23.32	4.62	8.83	4.62	1.33	7.81	2.02	3.19
no28	12.29	3.13	24.00	4.50	11.72	3.96	1.53	7.91	2.63	3.95
no29	15.59	3.67	21.57	4.49	5.98	4.17	1.49	6.74	2.00	2.57
no2	14.21	3.16	24.61	4.36	10.40	6.21	1.46	10.51	2.19	4.30
no30	11.76	3.11	17.60	4.02	5.84	4.33	0.81	5.45	1.11	1.11
no31	17.81	3.07	25.41	4.05	7.60	8.59	1.48	11.76	2.14	3.17
no32	14.07	2.58	18.81	4.05	4.74	5.76	1.13	6.69	1.52	0.93
no33	13.37	2.93	21.19	4.08	7.82	3.48	1.55	7.62	2.16	4.14
no34	13.19	3.42	18.98	3.93	5.79	4.87	1.67	6.92	1.70	2.05

no35	10.85	3.37	14.92	4.25	4.06	1.19	0.87	3.15	1.54	1.96
no36	8.33	1.73	17.80	4.16	9.47	2.95	0.97	5.55	1.47	2.59
no37	15.99	3.20	26.35	4.39	10.36	7.89	1.65	11.31	2.52	3.41
no38	12.51	3.47	19.50	4.08	6.99	2.83	1.32	6.14	1.84	3.32
no39	7.57	2.57	14.40	3.94	6.84	1.58	0.93	3.27	1.43	1.69
no3	9.65	2.56	18.95	4.43	9.31	1.98	1.04	4.07	1.68	2.08
no40	9.44	2.81	17.18	4.10	7.74	3.09	1.06	5.21	1.65	2.12
no41	14.91	3.53	20.91	4.48	6.00	4.99	1.08	7.49	1.70	2.50
no42	12.05	3.52	22.44	4.50	10.39	3.44	1.44	7.42	2.43	3.99
no43	14.07	3.64	21.99	4.15	7.92	5.00	1.23	8.29	1.82	3.29
no44	14.09	3.27	22.28	4.14	8.19	4.89	1.60	8.39	2.40	3.50
no45	12.67	3.46	22.20	4.33	9.53	3.72	1.36	7.52	2.17	3.80
no46	11.25	3.09	19.16	4.32	7.92	3.77	1.27	5.51	1.73	1.74
no47	14.02	3.71	23.03	4.43	9.01	5.23	1.63	8.38	2.18	3.16
no48	13.03	2.67	24.04	4.48	11.01	5.13	1.30	8.14	2.00	3.01
no49	11.49	3.77	18.51	4.19	7.02	2.76	1.20	5.04	1.90	2.28
no4	10.74	3.02	18.92	4.21	8.18	3.25	1.37	6.14	1.87	2.89
no50	12.19	3.13	19.72	4.34	7.53	4.10	1.34	5.87	1.83	1.77
no5	15.97	3.08	19.10	3.96	3.13	7.55	0.92	8.47	1.27	0.92
no6	11.44	2.95	16.57	4.42	5.13	2.95	1.12	4.43	1.40	1.48
no7	6.50	2.68	12.99	4.20	6.49	0.65	0.78	2.00	1.37	1.35
no8	15.01	2.70	20.66	3.93	5.65	6.69	1.18	8.40	1.70	1.70
no9	7.45	2.96	15.95	4.18	8.50	0.32	1.09	3.58	1.88	3.26
Mean	12.12	3.04	19.81	4.24	7.69	3.84	1.23	6.45	1.84	2.58

N.5 Pseudoknots (PK80)

	Boltzmann		Uniform			Boltzmann Optimized		Uniform Optimized		
Name	Mean	StdDev	Mean	StdDev	Δ	Mean	StdDev	Mean	StdDev	Δ_{opt}
no10	8.42	3.78	18.34	4.91	9.91	-0.53	1.83	3.19	2.18	3.73
no11	13.68	3.79	24.67	4.97	10.99	2.89	1.42	7.45	2.66	4.56
no12	15.00	3.06	22.26	4.84	7.26	4.23	1.48	6.91	2.13	2.69
no13	13.62	4.21	23.33	4.91	9.71	2.80	1.52	7.42	2.39	4.62
no14	11.77	4.14	24.61	5.11	12.84	1.16	1.71	7.11	2.62	5.95
no15	8.83	3.02	21.68	5.11	12.85	1.90	1.36	5.75	2.17	3.85
no16	16.73	3.84	25.45	4.93	8.72	4.85	1.71	8.75	2.46	3.90
no17	13.29	3.55	26.27	4.85	12.98	3.11	1.63	10.39	2.72	7.28
no18	15.42	3.88	25.61	4.75	10.19	4.07	2.03	7.90	2.57	3.83
no19	13.75	4.06	19.53	4.83	5.78	1.97	1.30	4.11	2.07	2.14
no1	17.32	4.56	25.64	5.06	8.32	4.59	1.58	8.62	2.43	4.03
no20	19.67	3.80	25.88	5.06	6.21	5.44	1.76	8.44	2.32	3.00
no21	19.96	3.54	31.00	4.98	11.05	9.09	1.76	14.15	2.57	5.06
no22	13.65	3.20	24.94	4.91	11.29	4.68	1.25	8.80	2.20	4.12
no23	12.98	3.15	20.28	4.68	7.29	4.54	1.42	6.15	1.81	1.61
no24	15.50	3.66	25.89	5.02	10.39	4.63	2.04	9.13	2.57	4.50
no25	14.84	4.18	27.83	5.01	12.99	4.02	2.19	9.80	2.75	5.78
no26	15.75	3.59	27.66	5.01	11.91	7.02	1.95	13.46	2.62	6.44
no27	19.33	3.55	31.95	4.84	12.63	9.79	2.04	15.87	2.86	6.08
no28	11.89	3.44	25.54	5.17	13.66	3.25	1.40	8.40	2.51	5.14
no29	17.60	4.42	23.52	5.13	5.92	4.03	1.52	6.91	2.17	2.87
no2	9.86	2.54	18.04	4.64	8.18	2.55	0.99	4.01	1.46	1.46

no30	12.37	3.49	23.07	4.83	10.70	3.17	1.74	7.65	2.41	4.49
no3	15.15	3.77	22.49	5.04	7.34	3.54	1.41	7.08	2.21	3.54
no4	11.10	2.79	24.80	4.95	13.70	2.01	1.46	8.97	2.64	6.96
no5	13.47	3.73	24.27	5.13	10.80	3.23	1.24	8.07	2.34	4.83
no6	4.89	3.44	17.34	4.71	12.45	-2.94	1.61	3.26	2.28	6.20
no7	12.98	2.54	25.77	4.84	12.79	4.66	1.43	9.68	2.09	5.03
no8	14.01	4.16	24.12	4.96	10.11	4.12	1.44	8.11	2.09	3.99
no9	11.37	3.97	19.71	4.71	8.33	1.24	1.40	4.04	2.16	2.80
Mean	13.81	3.63	24.05	4.93	10.24	3.64	1.59	7.99	2.35	4.35

N.6 LE80

						Boltzmann		Uniform		
	Boltzmann		Uniform			Optimized		Optimized		
Name	Mean	StdDev	Mean	StdDev	Δ	Mean	StdDev	Mean	StdDev	Δ_{opt}
PKB00002_PKB00004_0	8.78	2.66	14.95	3.72	6.16	2.24	0.91	4.09	1.31	1.84
PKB00005_PKB00015_0	9.69	3.10	12.97	3.27	3.28	2.91	0.92	4.13	1.06	1.23
PKB00008_PKB00031_0	10.62	2.40	12.56	3.33	1.94	2.91	0.78	4.11	0.99	1.19
PKB00010_PKB00066_0	7.07	2.06	12.79	3.32	5.73	2.64	0.80	3.63	1.07	1.00
PKB00012_PKB00268_0	8.77	2.59	12.54	3.38	3.77	2.77	0.90	3.69	1.10	0.92
PKB00030_PKB00045_0	6.31	1.82	12.63	3.44	6.32	1.79	0.73	3.39	1.10	1.59
PKB00047_PKB00069_0	15.24	2.54	19.86	3.99	4.62	6.69	1.26	7.73	1.48	1.05
PKB00048_PKB00265_0	10.02	3.36	18.64	4.04	8.62	3.77	1.09	8.21	1.74	4.44
PKB00050_PKB00128_0	11.11	3.77	16.42	4.42	5.30	1.90	1.06	3.72	1.46	1.82
PKB00052_PKB00107_0	6.72	2.72	16.17	4.45	9.45	1.43	1.07	3.82	1.43	2.40
PKB00057_PKB00072_0	12.13	2.89	19.82	4.42	7.68	5.34	1.24	7.70	1.72	2.37
PKB00068_PKB00129_0	15.42	3.03	23.78	4.67	8.36	7.01	0.98	8.84	1.49	1.83
PKB00070_PKB00244_0	9.22	2.84	18.45	4.09	9.23	2.49	1.35	5.57	1.78	3.07
PKB00078_PKB00106_0	4.45	2.08	18.46	4.51	14.01	0.12	1.00	4.62	1.85	4.50
PKB00080_PKB00132_0	8.13	2.10	13.01	3.47	4.89	2.20	0.79	3.40	1.17	1.20
PKB00088_PKB00127_0	7.00	3.06	19.26	4.94	12.26	0.89	1.03	4.53	1.82	3.64
PKB00098_PKB00232_0	13.16	3.07	21.53	4.33	8.37	5.38	1.20	8.00	1.64	2.62
PKB00131_PKB00205_0	11.86	2.78	22.53	4.12	10.67	5.17	1.21	7.67	1.79	2.50
PKB00139_PKB00141_0	12.53	2.91	19.13	4.33	6.61	4.14	1.00	6.46	1.58	2.31
PKB00142_PKB00231_0	14.46	3.53	19.98	4.91	5.52	4.51	1.50	6.29	1.87	1.79
PKB00143_PKB00233_0	9.67	3.05	17.78	4.36	8.11	2.96	1.12	5.47	1.66	2.52
PKB00148_PKB00218_0	12.06	3.00	22.54	4.75	10.48	5.54	1.38	8.79	2.07	3.26
PKB00175_PKB00259_0	9.21	2.09	17.94	4.14	8.73	3.99	0.87	6.22	1.42	2.24
PKB00179_PKB00280_0	16.60	2.06	23.05	5.63	6.45	3.32	1.43	4.88	1.73	1.55
PKB00180_PKB00212_0	13.09	2.12	21.19	4.75	8.09	5.23	1.45	7.23	1.91	2.01
PKB00190_PKB00266_0	6.13	2.85	12.98	3.52	6.85	0.21	0.97	2.20	1.34	1.99
PKB00207_PKB00213_0	6.60	3.00	12.03	3.39	5.43	1.14	0.83	2.56	1.08	1.42
PKB00211_PKB00239_0	12.92	3.59	22.54	4.81	9.61	4.82	1.44	8.91	2.10	4.09
PKB00222_PKB00305_0	16.14	2.91	26.40	5.73	10.26	6.64	1.66	9.07	2.12	2.43
PKB00224_PKB00281_0	10.61	3.09	14.72	3.58	4.11	3.01	1.05	4.34	1.28	1.33
PKB00230_PKB00273_0	7.91	2.41	15.08	3.80	7.16	3.89	1.31	6.56	1.82	2.67
PKB00248_PKB00257_0	9.72	3.12	18.37	4.20	8.64	6.44	1.31	11.39	2.71	4.95
PKB00263_PKB00270_0	12.00	2.17	18.83	4.19	6.83	4.53	1.09	6.34	1.58	1.81
PKB00269_PKB00272_0	12.93	3.37	16.19	3.78	3.26	4.10	1.20	6.19	1.75	2.09
Mean	10.54	2.77	17.80	4.17	7.26	3.59	1.11	5.88	1.59	2.28

References

[1] van Dijk T, van den Heuvel JP, Slob W. Computing treewidth with LibTW. University of Utrecht; 2006.

[2] Flamm C, Hofacker IL, Maurer-Stroh S, Stadler PF, Zehl M. Design of multistable RNA molecules. RNA (New York, NY). 2001 Feb;7:254–265.

[3] Bulatov AA, Dyer M, Goldberg LA, Jerrum M, Mcquillan C. The Expressibility of Functions on the Boolean Domain, with Applications to Counting CSPs. J ACM. 2013 Oct;60(5):32:1–32:36. Available from: http://doi.acm.org/10.1145/2528401.

[4] Weitz D. Counting independent sets up to the tree threshold. In: Proceedings of the 38th Annual ACM Symposium on Theory of Computing, Seattle, WA, USA, May 21-23, 2006; 2006. p. 140–149. Available from: http://doi.acm.org/10.1145/1132516.1132538.

[5] Cai JY, Galanis A, Goldberg LA, Guo H, Jerrum M, Štefankovič D, et al. # BIS-hardness for 2-spin systems on bipartite bounded degree graphs in the tree non-uniqueness region. Journal of Computer and System Sciences. 2016;82(5):690–711.

[6] Jerrum MR, Valiant LG, Vazirani VV. Random generation of combinatorial structures from a uniform distribution. Theoretical Computer Science. 1986;43(Supplement C):169 – 188. Available from: http://www.sciencedirect.com/science/article/pii/030439758690174X.

[7] Goldberg LA, Kelk S, Paterson M. The complexity of choosing an H-coloring (nearly) uniformly at random. SIAM Journal on Computing. 2004;33(2):416–432.
